# ACYP2 contributes to malignant progression of glioma through promoting Ca^2+^ efflux and subsequently activating c-Myc and STAT3 signals

**DOI:** 10.1186/s13046-020-01607-w

**Published:** 2020-06-09

**Authors:** Mengdan Li, Banjun Ruan, Jing Wei, Qi Yang, Mingwei Chen, Meiju Ji, Peng Hou

**Affiliations:** 1 Key Laboratory for tumor Precision Medicine of Shaanxi Province and Department of Endocrinology, The First Affiliated Hospital of Xi’an Jiaotong University, Xi’an, 710061 P.R. China; 2 Department of Endocrinology, The First Affiliated Hospital of Xi’an Jiaotong University, Xi’an, 710061 P.R. China; 3 Department of Respiratory and Critical Care Medicine, The First Affiliated Hospital of Xi’an Jiaotong University, Xi’an, 710061 P.R. China; 4 Center for Translational Medicine, The First Affiliated Hospital of Xi’an Jiaotong University, Xi’an, 710061 P.R. China

**Keywords:** Glioma, Acylphosphatase 2 (ACYP2), Calcium homeostasis, C-Myc, STAT3

## Abstract

**Background:**

Acylphosphatase 2 (ACYP2) is involved in cell differentiation, energy metabolism and hydrolysis of intracellular ion pump. It has been reported as a negative regulator in leukemia and a positive regulator in colon cancer, respectively. However, its biological role in glioma remains totally unclear.

**Methods:**

We performed quantitative RT-PCR (qRT-PCR), immunohistochemistry (IHC) and western blot assays to evaluate ACYP2 expression. The functions of ACYP2 in glioma cells were determined by a series of in vitro and in vivo experiments, including cell proliferation, colony formation, cell cycle, apoptosis, migration, invasion and nude mouse tumorigenicity assays. In addition, western blot and co-immunoprecipitation (Co-IP) assays were used to identify its downstream targets.

**Results:**

Knocking down ACYP2 in glioma cells significantly inhibited cell proliferation, colony formation, migration, invasion and tumorigenic potential in nude mice, and induced cell cycle arrest and apoptosis. Conversely, ectopic expression of ACYP2 in glioma cells dramatically promoted malignant phenotypes of glioma cells. Mechanistically, ACYP2 promoted malignant progression of glioma cells through regulating intracellular Ca^2+^ homeostasis via its interaction with PMCA4, thereby activating c-Myc and PTP1B/STAT3 signals. This could be effectively reversed by Ca^2+^ chelator BAPTA-AM or calpain inhibitor calpeptin.

**Conclusions:**

Our data demonstrate that ACYP2 functions as an oncogene in glioma through activating c-Myc and STAT3 signals via the regulation of intracellular Ca^2+^ homeostasis, and indicate that ACYP2 may be a potential therapeutic target and prognostic biomarker in gliomas.

## Background

Gliomas are the most common malignant tumor in the central nervous system, and the overall estimated annual incidence for gliomas ranges from 4.67 to 5.73 per 100,000 individuals [1–3]. They originate from brain interstitial cells and hold the characteristics of diffuse infiltrative growth, no definite boundaries and highly invasive. Glioblastoma (GBM), which is the deadliest subtype, accounts for about 50% of diffuse gliomas. The median survival durations of GBM patients is 14–17 months and ~ 12 months in contemporary clinical trials [4–6] and population-based studies [7, 8], respectively. Thus, it is of great importance to uncover potential therapeutic targets and prognostic biomarkers, and develop effective treatment strategies.

Acylphosphatase (ACYP) is a small cytosolic enzyme widely present in vertebrate tissues. Two isoenzymatic forms are coded by *ACYP1* and *ACYP2*, and termed “muscle type” (MT) and “common type” (CT), respectively. They share a highly conserved amino acid sequence identity. The MT isoform is prevalently expressed in skeletal muscle and heart, while the CT isoform is mainly expressed in erythrocytes, brain and testis [9]. These two enzymes catalyze the hydrolysis of the carboxyl-phosphate bond present in metabolites like 1,3-biphosphoglycerate, carbamoyl phosphate and also in proteins, such as the β-aspartyl phosphate intermediates formed during the actions of the Na^+^, K^+^-ATPase of erythrocyte plasma membrane and the Ca^2+^-ATPase of both erythrocyte membrane and heart sarcolemma [10–12]. ACYP has been reported to be expressed exclusively in human metastatic colorectal lines, suggesting that it may be linked to metastatic phenotype [13]. In addition, ACYP has also been proven to be involved in differentiation of human erythroleukemia K562 cell line [14], and ectopic expression of ACYP2 can induce cell apoptosis in HeLa cells [15]. Altogether, the above observations indicate that ACYP may be involved in tumor initiation and progression; however, there are no studies available to determine their role in gliomas.

In this study, we observe that ACYP2 is significantly upregulated in gliomas, and find a significant association of increased expression of ACYP2 with poor patient survival in low-grade glioma patients. Functional studies demonstrate that ACYP2 acts as an oncogenic function in glioma cells through regulating intracellular Ca^2+^ homeostasis and subsequently activating c-Myc and STAT3 signals.

## Materials and methods

### Clinical samples

A total of 52 frozen surgical gliomas and 24 normal brain tissues from cerebral contusion and laceration patients were randomly obtained from the First Affiliated Hospital of Xi’an Jiaotong University. A part of the above tissues were taken, fixed in 10% formalin and embedded in paraffin for immunohistochemical analysis. None of these patients received any preoperative chemotherapy, radiotherapy or other biological therapy, and all patients signed an informed consent before the surgery. All of the tissues were histologically examined by two senior pathologists at the Department of Pathology of the Hospital based on World Health Organization (WHO) criteria, and study protocol was approved by the Institutional Review Board and Human Ethics Committee of the First Affiliated Hospital of Xi’an Jiaotong University.

### RNA extraction and quantitative RT-PCR (qRT-PCR)

RNA isolation, cDNA synthesis and RT-qPCR was carried out as described previously [16]. The mRNA expression of the indicated genes was normalized to *18S* rRNA, and each sample was run in triplicate. The primer sequences were summarized in Additional file 1: Table S1.

### Cell lines and drug treatments

Human glioma cell lines U251, SHG44, A172, U87, BT325 and SF295 were provided by Cell Bank of the Zhongshan University. A172 and BT325 was provided by Kunming Cell Bank of The Chinese Academy of Sciences. Cells were all routinely cultured at 37 °C in DMEM medium with 10% fetal bovine serum (FBS). All cell lines used in this study were authenticated by short tandem repeat (STR) analysis in Genesky Co. Ltd. (Additional file 1: Table S2), and the results was completely consistent with previous studies [16] and database (Cellosaurus: https://web.expasy.org/cellosaurus/). In some experiments, cells were treated with 100 μM cell-permeable c-Myc-Max dimerization inhibitor 10,058-F4 (Selleck Chemicals) for 48 h to inhibit transcriptional activity of c-Myc. Cells were treated with 5 μM BAPTA-AM (Selleck Chemicals) for 6 h to chelate intracellular Ca^2+^. Cells were treated with 10 μM calpeptin (Selleck Chemicals) for 12 h to block calpain activity. Cells were treated with 10 μM sodium orthovanadate (Na_3_VO_4_) for 1 h to inhibit PTP1B activity. The same volume of the vehicle was used as the control.

### siRNAs, expression plasmids and lentivirus transfection

Oligonucleotides of siRNAs targeting ACYP2, PMCA4 and PTP1B were obtained from Gene Pharma (Shanghai, China) and Ribobio (Guangzhou, China), respectively. The sequences were presented in Additional file 1: Table S3. Cells were transfected at 50% confluence using Lipofectamine 2000 (Invitrogen, Grand Island, NY) according to the instructions of the manufacturer, with a final siRNA concentration of 50 nM. All silencing experiments were carried out in triplicate. Two oligonucleotides with maximal knockdown efficiency were selected among three different sequences.

Open reading frame (ORF) of ACYP2 with stop codon was amplified and then cloned into pcDNA3.1(−) mammalian expression vector, termed pcDNA3.1(−)-ACYP2. The primer sequences were shown in Additional file 1: Table S4. Cells were transfected with the indicated constructs at 70% confluence using X-treme GENE HP DNA Transfection Reagent (Invitrogen, Grand Island, NY) according to the instructions of the manufacturer. Lentivirus encoding shRNA targeting ACYP2 and control lentivirus were obtained from HanBio Biotechnology Co., Ltd. (Shanghai, China). Cells were transfected at 50% confluence with a final lentivirus multiplicity of infection (MOI) of 50–100 according to the instructions of the manufacturer. Cells stably knocking down ACYP2 were selected by puromycin.

### In vitro functional studies

Cell proliferation was evaluated by MTT assay. Soft-agar assay was performed to assess colony formation ability. Cell apoptosis was evaluated by flow cytometer. Cell migration and invasion abilities were evaluated by transwell chambers. Each experiment was run in triplicate.

### Western blot and co-immunoprecipitation (co-IP) assays

The detailed procedures were carried out as described previously [17]. Antibody information was summarized in Additional file 1: Table S5.

### Measurement of intracellular Ca^2+^

The intracellular cytosolic-free Ca^2+^ concentration was measured under the confocal microscope (Leica) or flow cytometer by using the Ca^2+^-sensitive dye Fluo-4 AM (Molecular Probes, Invitrogen). For the former, cells were seeded in Glass Bottom Culture Dishes (MatTek Corporation) before transfection with siRNAs targeting ACYP2 or control siRNA. After 48 h, the medium was removed, and cells were loaded with Fluo-4 AM (1 μmol/L) for 30 min at 37 °C with gentle shaking. Next, cells were washed and incubated for 20 min at 37 °C prior to experiments. Fluorescence intensity was determined at 494 nm excitation and 516 nm emission. For flow cytometer analysis, cells were trypsinized, washed and placed in Eppendorf tubes at 1 × 10^6^/mL, and incubated with Fluo-4 AM (1 μmol/L) at 37 °C for 30 min. Data were expressed as fluorescence intensity.

### Measurement of intracellular calpain

Activated calpain in the protein extract was measured using a calpain activity assay kit (Abcam, Cat. # ab65308). Protein lysates were extracted following the manufacturer’s instruction. Briefly, cytosolic protein extracts of glioma cells were prepared with the extraction buffer which prevents the auto-activation of calpain during the extraction procedure. The calpain activity was quantified by the measurement of fluorescence reading at λ max =505 nm using calpain substrate Ac-LLY-AFC. The activity was represented as relative fluorescence units (RFU)/mg protein.

### Animal studies

Four-week-old male athymic nude mice were purchased from SLAC laboratory Animal Co., Ltd. (Shanghai, PR. China) and housed in a specific pathogen-free (SPF) environment. The mice were randomly divided into four groups (5 mice per group). SF295 cells stably knocking down ACYP2 or control cells (1 × 10^7^) were implanted in nude mice to establish tumor xenografts. From day 3 post-injection, BAPTA-AM (9 mg/kg), calpeptin (2 mg/kg) or vehicle were administered by intraperitoneal injection in 1.0% DMSO, and tumor size was measured every 2 days. Tumor volumes were calculated by the formula (length × width^2^ × 0.5). Dosing was daily for 9 consecutive days. At the end of experiments, xenograft tumors were harvested and weighted. In addition, a part of tumor tissues were fixed in 15% formalin for 24 h, embedded in paraffin, and sectioned at 4 μm until use. Proliferation ability of xenograft tumors was assessed by quantification with Ki-67 immunohistochemistry. All animals’ experimental procedures were approved by the Laboratory Animal Center of Xi’an Jiaotong University.

### Immunohistochemistry (IHC)

The IHC assay was carried out to evaluate protein expression of ACYP2, c-Myc, NCL, phosphorylated STAT3 (Tyr705) and Ki67. The detailed protocol was carried out as described previously [18].

### Statistical analysis

Gene expression in tumor tissues and control subjects were compared by the unpaired *t* test. Survival curves were constructed according to the Kaplan-Meier method, and statistical analysis was performed using the Log-rank test. All statistical analyses were performed using the SPSS statistical package (16.0, Chicago, IL). *P* < 0.05 were considered statistically significant.

## Results

### Increased expression of ACYP2 is associated with poor survival in low-grade glioma (LGG) patients

**Fig. 1:**
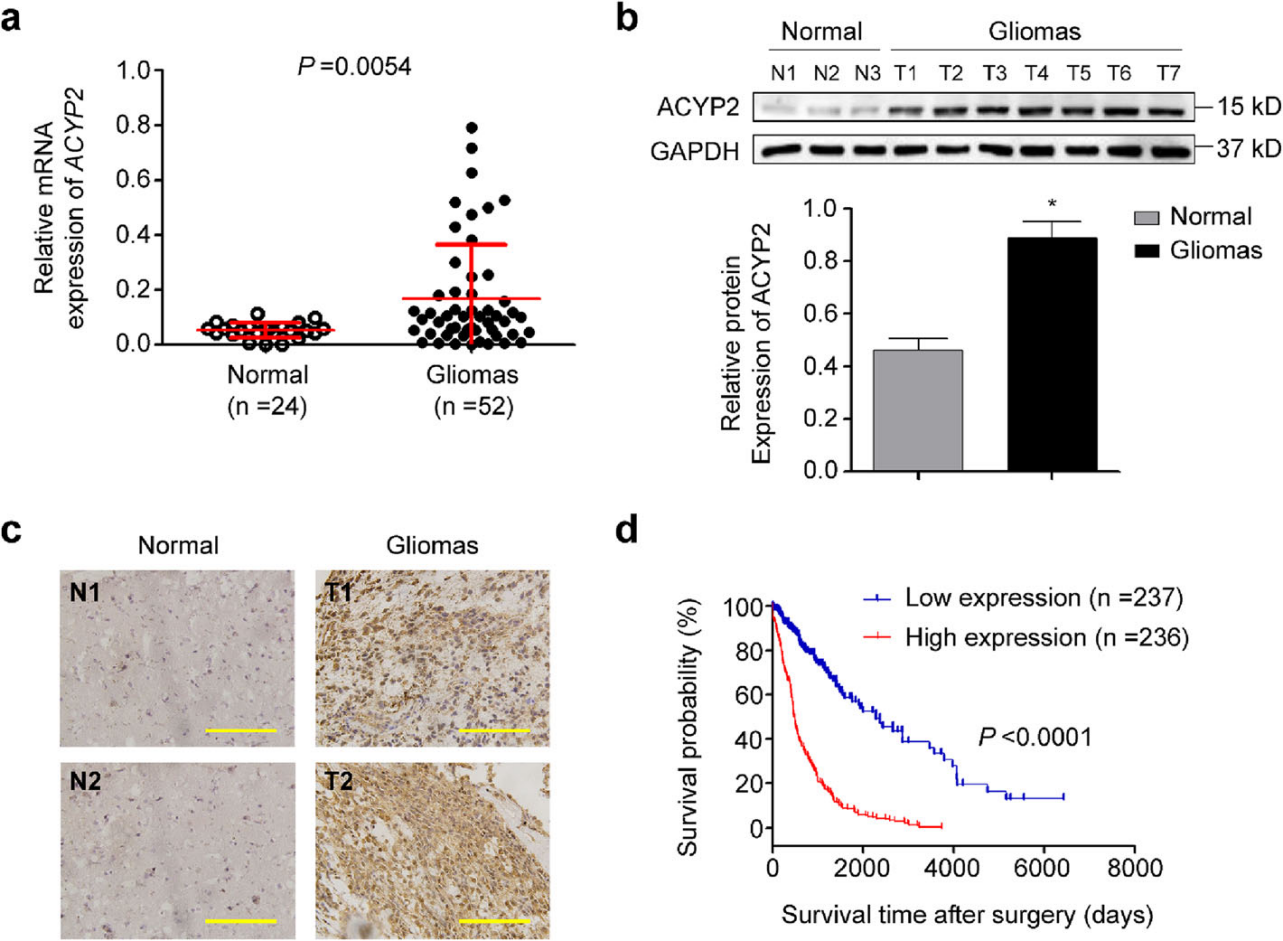
Association of increased expression of ACYP2 with poor survival in low-grade glioma (LGG) patients. **a**, mRNA expression of ACYP2 in normal brain tissues (*n* = 24) and glioma tissues (*n* = 52) was analyzed by qRT-PCR assay. Its expression was normalized to *18S* rRNA levels. **b**, Protein expression of ACYP2 in normal brain tissues (N, *n* = 3) and glioma tissues (T, *n* = 7) was analyzed by western blot analysis (upper panel). GAPDH was used as a loading control. Quantitative illustration of ACYP2 protein levels are shown in the lower panel. **c**, Representative images showing immunohistochemistry (IHC) staining of ACYP2 on histologic slides of normal brain tissues and glioma tissues. **d**, A significant association of increased expression of ACYP2 with poor survival in LGG patients (*n* = 473; data from TCGA dataset). *, *P* < 0.05

We first examined mRNA and protein expression of ACYP2 in 52 gliomas and 24 normal brain tissues (control subjects) by qRT-PCR, immunohistochemistry and western blot assays. As shown in Fig. 1a-c, ACYP2 expression was significantly elevated in glioma tissues in comparison with control subjects at both mRNA and protein levels. Next, through analyzing the Cancer Genome Atlas (TCGA) dataset, we found that increased expression of *ACYP2* was clearly associated with poor survival in low-grade glioma (LGG) patients (HR =0.23 with log-rank *P* < 0.0001; Fig. 1d), but not in glioblastoma (GBM) patients (data not shown).

### ACYP2 promotes glioma cell growth in vitro

**Fig. 2:**
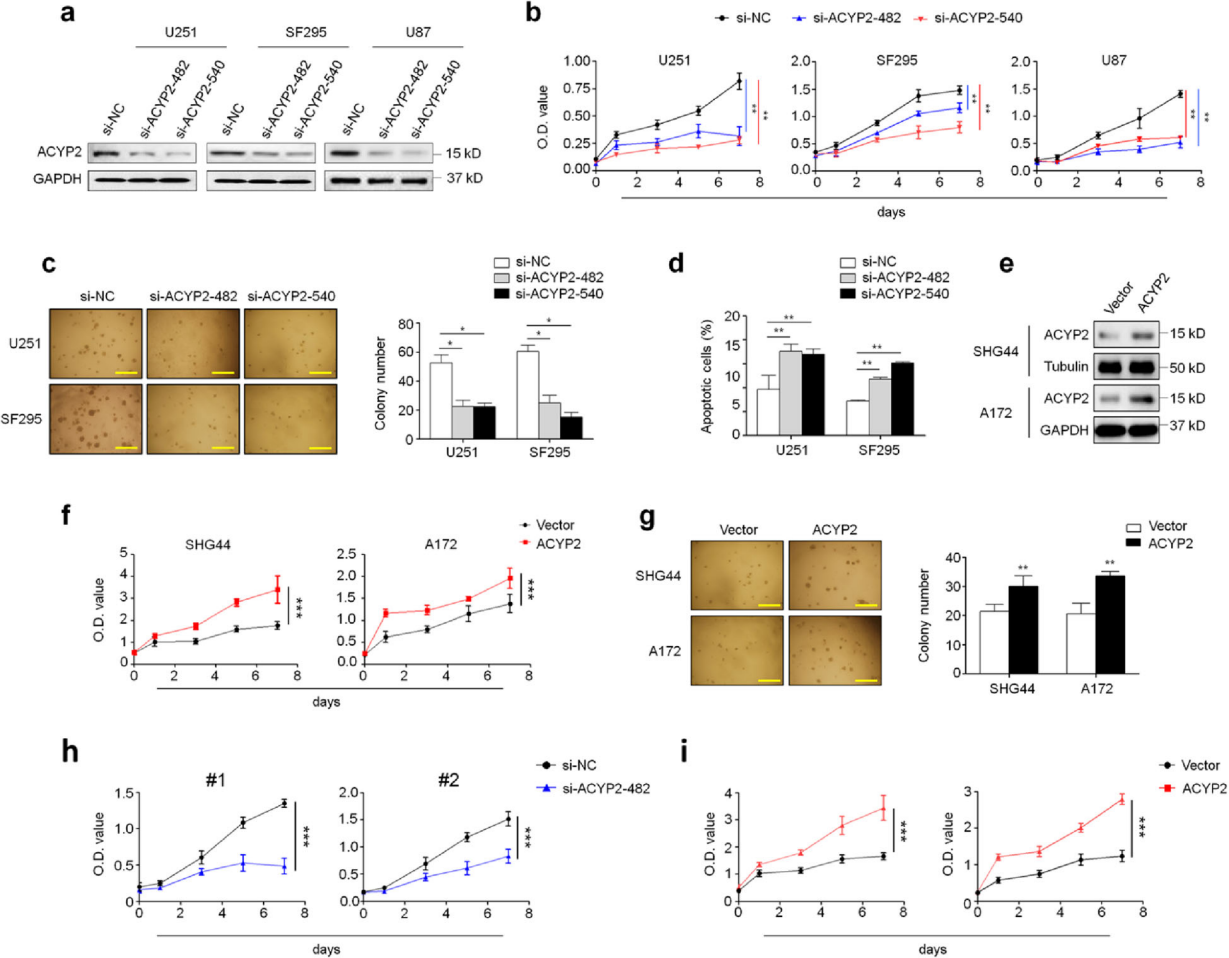
Growth-promoting effect of ACYP2 on glioma cells in vitro. **a**, Western blot analysis was carried out in U251, SF295 and U87 cells to confirm ACYP2 knockdown by two different siRNAs (si-ACYP2–482 and − 540). GAPDH were used as a loading control. **b**, ACYP2 knockdown significantly inhibited cell proliferation in comparison with the control. **c**, Knocking down ACYP2 in the indicated cells significantly inhibited colony formation. Representative images showing colony formation in the left panel, and quantitative analysis was shown in the right panel. **d**, Cell apoptosis in the indicated cells were measured by flow cytometry. The data were presented as mean ± SD (*n* = 3). **e**, Ectopic expression of ACYP2 was confirmed by western blot analysis. Tubulin and GAPDH were used as loading controls. **f** and **g**, Ectopic expression of ACYP2 significantly promoted glioma cell proliferation and colony formation. The effects of knockdown (**h**) and ectopic expression (**i**) of ACYP2 on the proliferation of of primary human glioma cells. The data were presented as mean ± SD (*n* = 3). Scale bar: 200 μm; *, *P* < 0.05; **, *P* < 0.01; ***, *P* < 0.001

A series of in vitro experiments were performed to determine biological role of ACYP2 in malignant phenotypes of glioma cells. First, we used two different siRNAs (si-ACYP2–482 and − 540) to knock down ACYP2 expression in U251, SF295 and U87 cells, and validated their knockdown efficiency by qRT-PCR (Additional file 2: Figure S1) and western blot analysis (Fig. 2a). The results showed that ACYP2 knockdown in U251 and SF295 cells significantly inhibited cell proliferation (Fig. 2b) and colony-forming ability on soft agar (Fig. 2c) compared to the control. In addition, we also tested the effect of ACYP2 depletion on the apoptosis of glioma cells. As shown in Fig. 2d, ACYP2 knockdown in these cells dramatically induced cell apoptosis in comparison with the control (9.7 ± 2.9% vs. 17.0 ± 1.1% in U251 cells, *P* = 0.004; 7.3 ± 0.1% vs.15.2 ± 0.2% in SF295 cells, *P* = 0.005). On the other hand, ectopic expression of ACYP2 in SHG44 and A172 cells clearly promoted cell proliferation and colony-forming ability in comparison with the control (Fig. 2e-g). To further validate the above conclusions, we knocked down or ectopically expressed ACYP2 in primary tumor cells from two glioma patients, and demonstrated that ACYP2 knockdown significantly inhibited cell proliferation, while ectopic expression of ACYP2 strongly promoted cell proliferation (Fig. 2h and i). Taken together, these data support the oncogenic role of ACYP2 in glioma cells.

### ACYP2 promotes glioma cell migration and invasion

The effect of ACYP2 on cell migration and invasion was next assessed in three glioma cell liens. As shown in Additional file 2: Figure S2a, ACYP2 knockdown in U251, SF295 and U87 cells significantly inhibited cell migration in comparison with the control. In addition, we also found that ACYP2-knockdown cells exhibited a relatively low ability of cell invasion compared to control cells (Additional file 2: Figure S2b). On the other hand, ectopic expression of ACYP2 in SHG44 and A172 cells clearly promoted cell migration and invasion in comparison with the control (Additional file 2: Figure S3). These results indicate that there is a strong link between aberrant expression of ACYP2 and metastatic phenotypes of glioma cells.

### ACYP2 promotes malignant phenotypes of glioma cells through enhancing Ca^2+^ efflux

**Fig. 3:**
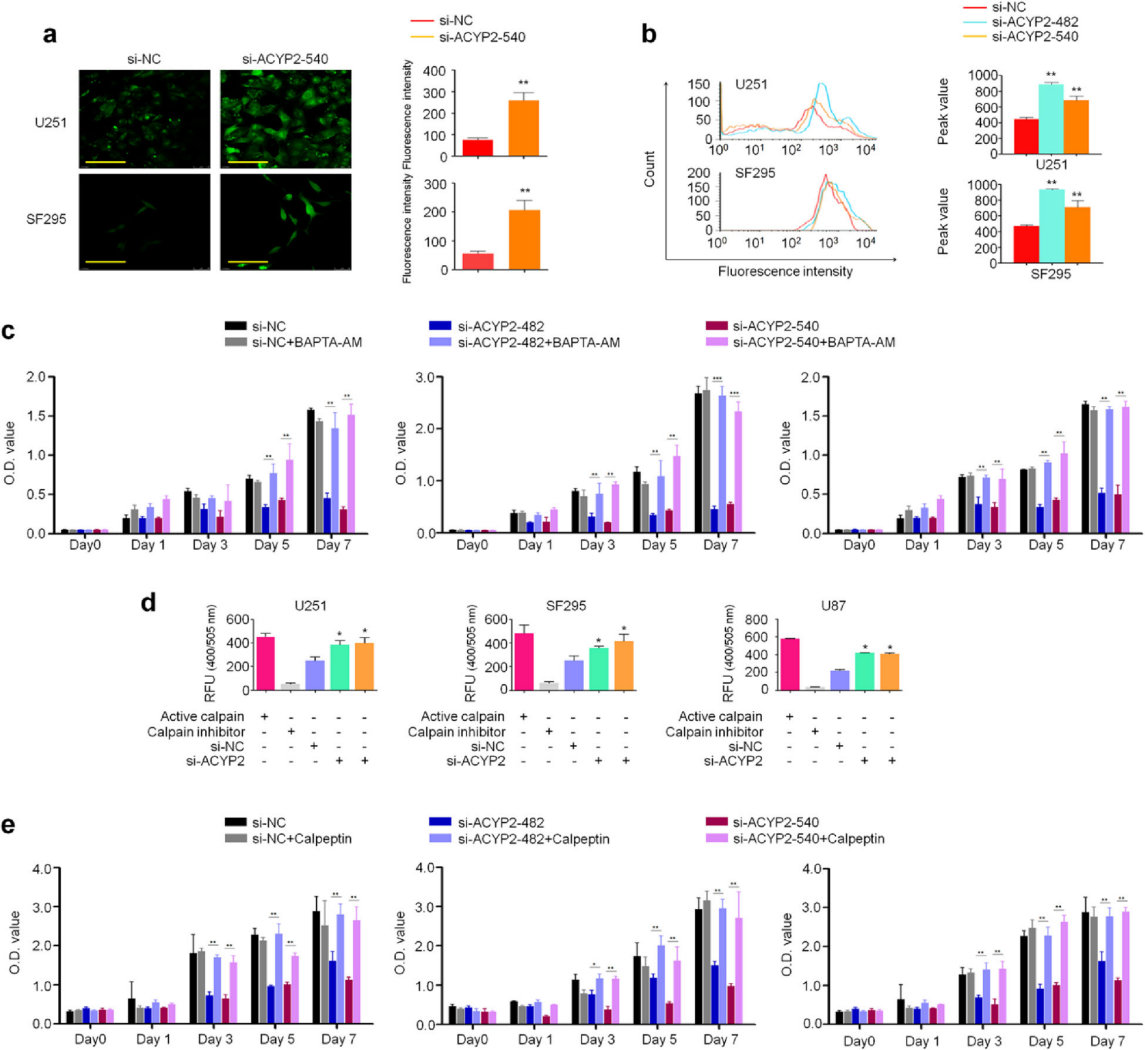
ACYP2 promotes glioma cell proliferation through altering intracellular Ca^2+^ homeostasis and calpain activity. **a**, Representative images showing free intracellular Ca^2+^ in the indicated cells under the confocal microscope (left panel). Green color represents staining of free intracellular Ca^2+^. Histogram represents mean ± SD of the fluorescence intensity from five microscopic fields in each group, as shown in right panel. Scale bars, 200 μm. **b**, Free intracellular Ca^2+^ measured by flow cytometer was shown in the middle and right panels. The data were presented as mean ± SD (*n* = 3). **c**, Cells were transfected with the indicated siRNAs and treated with the vehicle or 5 μM BAPTA-AM for 6 h, the MTT assay was then carried out to evaluate their effect on cell proliferation. The data were presented as mean ± SD (*n* = 3). **d**, Calpain activity was measured in the indicated cells using a calpain activity assay kit. Active Calpain I and calpain inhibitor Z-LLY-FMK were used as positive and negative control, respectively. Data are presented as mean ± SD (*n* = 3). **e**, Cells were transfected with the indicated siRNAs and treated with the vehicle or 10 μM calpeptin for 12 h, and the MTT assay was then carried out to evaluate their effect on cell proliferation. The data were presented as mean ± SD (*n* = 3). *, *P* < 0.05; **, *P* < 0.01; ***, *P* < 0.001

ACYP can catalyze the hydrolysis of not only low molecular weight substrates, such as carbamoyl phosphate and succinoyl phosphate, but also the acylphosphorylated intermediates of P-type ATPases, including plasma membrane Ca^2+^-ATPase (PMCA) [19]. To reveal molecular mechanisms underlying oncogenic role of ACYP2 in glioma cells, we first tested the effect of ACYP2 on intracellular Ca^2+^ concentration. As shown in Fig. 3a, under the fluorescence microscope, ACYP2 depletion in U251 and SF295 cells leaded to a significant increase of Ca^2+^ fluorescence intensity in the cytoplasm of tumor cells relative to the control. As supported, flow cytometry analysis showed rightward shifting of peak value in ACYP2-knockdown cells, indicating the increase of intracellular Ca^2+^ concentration (Fig. 3b). Besides, considering that endoplasmic reticulum (ER) Ca2+ pumps also belong to the P-type pump family, and there is evidence demonstrating that ACYP regulates Ca2 + −ATPase activity and Ca2+ transport on cardiac and skeletal ER [20, 21], thus we next tested the effect of ACYP2 on calcium pool in ER by loading the cells with the Ca2 + −sensitive fluorescent probe Mag-fura 2, which has relatively high Ca2+ affinity for ER. As expected, we found that ACYP2 knockdown in U251 and SF295 cells significantly decreased Ca2+ fluorescence intensity in ER relative to the control (Additional file 2: Figure S4). Given the above, we speculated that ACYP2 might act as its oncogenic role by altering intracellular Ca^2+^ homeostasis. To prove this, we treated U251, SF295 and U87 cells with 5 μM BAPTA-AM, a highly selective Ca^2+^ chelator, for 6 h. The results showed that inhibitory effect of ACYP2 knockdown on cell proliferation was effectively reversed upon BAPTA-AM treatment (Fig. 3c). This was also supported by cell migration assay (Additional file 2: Figure S5).

Calpains are a family of cytosolic cysteine proteinases whose enzymatic activities depend on Ca^2+^. Members of the calpain family are believed to function in various biological and pathological processes, including tumorigenesis [22–24]. Thus, we suppose that ACYP2 may alter the activity of calcium-dependent calpains through modulating intracellular Ca^2+^ homeostasis. This will lead to changes of proteolytically cleavage of transcription factors in the cytoplasm and abnormal activation of some major signaling pathways, thereby promoting malignant progression of glioma. To prove this, we used Calpain Activity Assay Kit to determine the effect of ACYP2 depletion on calpain activity in U251, SF295 and U87 cells. Active calpain and calpain inhibitor calpeptin were used as positive and negative control, respectively. As shown in Fig. 3d, ACYP2 knockdown caused a significant increase of calpain activity in these cells in comparison with the control. Next, we treated the above glioma cell lines with 10 μM calpeptin for 12 h, and expectedly found that inhibitory effect of ACYP2 knockdown on cell proliferation was reversed upon calpeptin treatment (Fig. 3e). Similarly, calpeptin treatment also effectively reversed inhibitory effect of ACYP2 knockdown on cell migration in comparison with the control (Additional file 2: Figure S6). These data indicate that ACYP2 acts as an oncogenic function in glioma cells through altering intracellular Ca^2+^ homeostasis and calpain activity.

In addition to Ca2+/calpain signaling pathway, the activation of NFATc1, 2, 3 and 4 is also intracellular Ca^2+^-dependent, and there is evidence showing that NFATc1 as a key transcription factor can promote the migration and adhesion of GBM cells [25]. Thus, we also determined the effect of ACYP2 knockdown on the activity of NFATc1 by western blot and immunofluorescence assays. The results showed that ACYP2 knockdown almost did not affect NFATc1 expression in both cytoplasm and nucleus, cytoplasm-to-nucleus translocation of NFATc1, and the expression of its downstream target PTGS2 (Additional file 2: Figure S7). It has also been reported that intracellular Ca2+ can regulate NF-kB transcriptional activity in human aortic endothelial cells [26]. This was supported by our data that ACYP2 knockdown clearly decreased the expression of downstream targets of NF-kB signaling, Bcl-xL and Bcl-2, in U251 and SF295 cells (Additional file 2: Figure S8). The above data indicate that ACYP2 may be involved in various intracellular Ca2 + −related signaling pathways, and we mainly focused on Ca2+/calpain-related signaling pathway in this study.

### ACYP2 activates c-Myc and STAT3 signals by regulating intracellular Ca^2+^ homeostasis and calpain activity

**Fig. 4:**
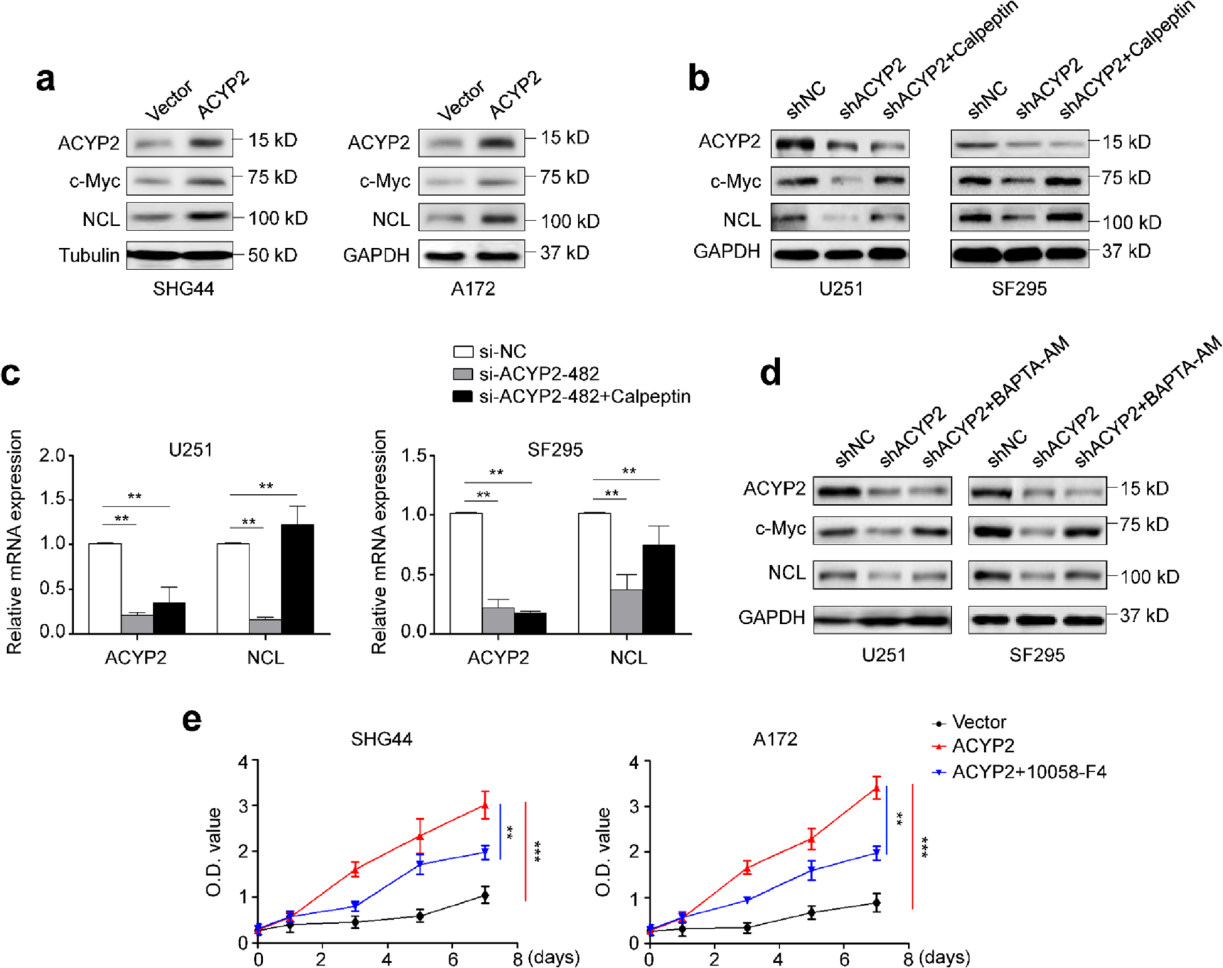
Transcriptional activation of c-Myc by ACYP2 in glioma cells through regulating intracellular Ca^2+^ homeostasis and calpain activity. **a**, Western blot analysis was carried out to determine the effect of ectopic expression of ACYP2 in SHG44 and A172 cells on the levels of transcriptionally active c-Myc and its downstream target NCL. Tubulin and GAPDH were used as loading controls. Cells transfected with the indicated shRNAs were treated with the vehicle or 10 μM calpeptin for 10 h. **b**, Western blot analysis was performed to evaluate their effect on protein expression of c-Myc and its target NCL. **c**, qRT-PCR assay was used to evaluate their effect on mRNA levels of *ACYP2* and *NCL*. GAPDH was used as a loading control in western blot analysis. *18S* rRNA was used as a reference gene in qRT-PCR assay. **d**, Cells transfected with the indicated shRNAs were treated with the vehicle or 5 μM BAPTA-AM for 6 h. Western blot analysis was then performed to investigate their effect on the expression of c-Myc and its downstream target NCL. GAPDH was used as a loading control. **e**, The indicated cells were treated with the vehicle or 100 μM 10,058-F4 for 48 h, and the MTT assay was then used to evaluate their effect on cell proliferation. The data were presented as mean ± SD (*n* = 3). **, *P* < 0.01; ***, *P* < 0.001

Calpain is a proteinase with relatively poor specificity and functions in many cellular pathways through controlled proteolysis of various substrates [27]. Thus, we speculate that ACYP2/Ca2+/calpain signaling axis may act on some key transcription factors and affect their activity, thereby contributing to malignant phenotypes of glioma cells. There is evidence reporting that c-Myc proteins can be proteolytically cleaved by Ca2 + −dependent calpains at lysine 298, and this generates Myc-nick, a transcriptionally inactive cleavage product of c-Myc oncoproteins [28]. To prove this, we examined c-Myc levels in glioma cells ectopically expressing or knocking down ACYP2 by western blot using antibody against C terminus of c-Myc (9E10), which recognizes only full-length c-Myc [29]. As shown in Fig. 4a, ectopic expression of ACYP2 clearly elevated the levels of transcriptionally active c-Myc and its downstream target NCL (nucleolin) in SHG44 and A172 cells. Conversely, ACYP2 knockdown decreased their levels, while this effect could be effectively reversed by calpeptin treatment (Fig. 4b). This was also supported by the results of qRT-PCR assay (Fig. 4c). Besides, ACYP2 knockdown also significantly down-regulated the expression of its other two downstream targets, E2F2 and cyclin E, in U251 and SF295 cells compared to the control (Additional file 2: Figure S9). Similarly, BAPTA-AM treatment also reversed inhibitory effect of ACYP2 depletion on transcriptional activity of c-Myc (Fig. 4d). Next, we attempted to determine the potential role of c-Myc in oncogenic function of ACYP2 in glioma cells. The results showed that promoting effect of ACYP2 on glioma cell proliferation could be partially reversed by the treatment of c-Myc inhibitor 10,058-F4 (Fig. 4e).

**Fig. 5:**
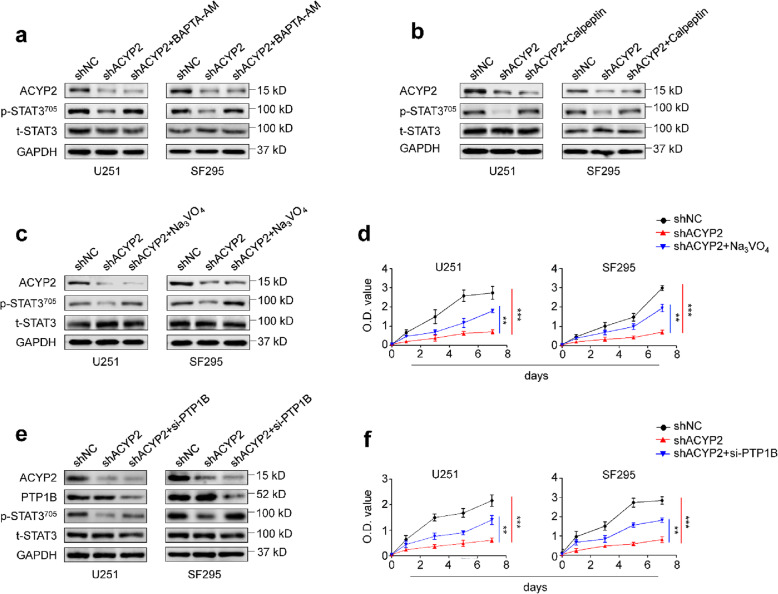
ACYP2 promotes STAT3 phosphorylation in glioma cells through regulating intracellular Ca^2+^ homeostasis and calpain activity. Cells transfected with the indicated shRNAs were treated with 5 μM BAPTA-AM for 6 h (**a**), 10 μM calpeptin for 10 h (**b**), 10 μM Na3VO4 for 1 h (**c**), or the vehicle. Western blot analysis was then performed to test their effect on STAT3 phosphorylation. GAPDH was used as a loading control. **d**, Cells transfected with the indicated shRNAs were treated with the vehicle or 10 μM Na3VO4 for 1 h, and the MTT assay was then carried out to evaluate their effect on cell proliferation. The data were presented as mean ± SD (*n* = 3). Cells stably knocking down ACYP2 and control cells were transfected with siRNA targeting PTP1B or not. **e**, Western blot analysis was then performed to evaluate their effect on STAT3 phosphorylation. **f**, MTT assay was performed to assess their effect on cell proliferation. The data were presented as mean ± SD (*n* = 3). **, *P* < 0.01; ***, *P* < 0.001

Signal transducer and activator of transcription 3 (STAT3) has been reported to be aberrantly activated in gliomas, and be identified as a potential therapeutic target [30]. There is evidence showing that STAT3 activation is dependent on Ca2+ signal in breast cancer cells, and phosphorylation of STAT3 at Tyr705 by EGF can be substantially inhibited upon intracellular Ca2+ chelation [31]. Thus, we suppose that ACYP2 may promote phosphorylation and activity of STAT3 through modulating Ca2+/calpain signaling axis, contributing to malignant progression of glioma cells. The results showed that ACYP2 knockdown dramatically decreased the levels of STAT3 phosphorylation (Tyr705) in comparison with the control, while this effect could be effectively reversed by BAPTA-AM or calpeptin treatment (Fig. 5a and b). As expected, ACYP2 knockdown dramatically decreased the expression of its two downstream targets, c-Fos and c-Jun, in U251 and SF295 cells compared to the control (Additional file 2: Figure S9).

It should be noted that calpain-deficient breast cancer cells exhibit impaired invadopodia formation, which can be rescued by ectopic expression of protein tyrosine phosphatase 1B (PTP1B) [32]. In addition, PTP1B cleavage by calpain is considered as a general feature of platelet agonist-induced aggregation [33]. Thus, we speculate that ACYP2 may activate STAT3 by regulating Ca2+/calpain/PTP1B signaling axis. To prove this, we used the PTP1B inhibitor sodium aluminate (Na3VO4) to treat U251 and SF295 cells stably knocking down ACYP2 and control cells. As shown in Fig. 5c and d, Na3VO4 could reverse inhibitory effect of ACYP2 depletion on STAT3 phosphorylation and cell proliferation. Considering that Na3VO4 is a non-specific PTP1B inhibitor, we next used siRNA approach to knock down PTP1B, and observed the similar findings, as shown in Fig. 5e and f. Finally, we treated SHG44 and A172 cells with 1 or 5 μM STAT3 inhibitor Stattic, and found that Stattic partially reversed promoting effect of ACYP2 on cell proliferation in a dose-dependent manner (Additional file 2: Figure S10). The above findings indicate that ACYP2 can activate c-Myc and STAT3 signals through modulating intracellular Ca^2+^ homeostasis and calpain activity, thereby contributing to malignant phenotypes of glioma cells.

### ACYP2 exerts its oncogenic function through interacting with PMCA4

**Fig. 6:**
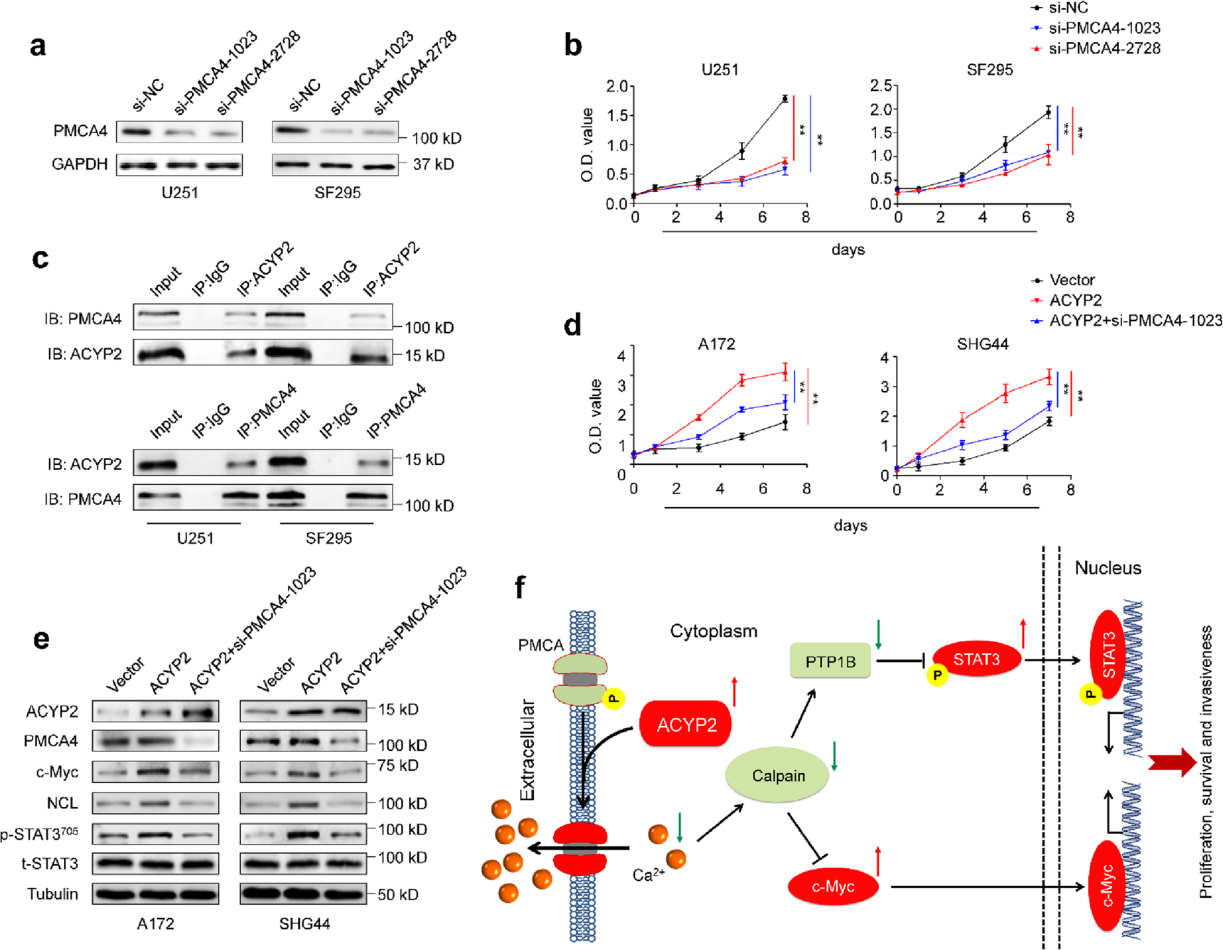
ACYP2 promotes malignant phenotypes of glioma cells through interacting with PMCA4. **a**, Western blot analysis was performed in glioma cells to confirm PMCA4 knockdown by two different siRNAs (si-PMCA4–1023 and − 2728). GAPDH was used as a loading control. **b**, PMCA4 knockdown significantly inhibited glioma cell proliferation in comparison with the control. **c**, Co-IP was carried out in glioma cells to determine the interaction between ACYP2 and PMCA4 using antibodies against ACYP2 (upper panel) and PMCA4 (lower panel). Cells ectopically expressing ACYP2 and control cells were transfected with siRNA targeting PMCA4 or not. **d**, MTT assay was carried out to evaluate their effect on cell proliferation. **e**, Western blot analysis was used to investigate their effect on the activities of c-Myc and STAT3. Tubulin was used as a loading control. **f**, A schematic model of ACYP2 promoting malignant progression of glioma. Briefly, increased expression of ACYP2 interacts with and activates PMCA4, increasing the efficiency of the transport and promoting Ca^2+^ efflux. Lower free intracellular Ca^2+^ can decrease calpain activity, reducing the cleavage of c-Myc and PTP1B and subsequently enhancing transcriptional activities of c-Myc and STAT3. This will ultimately contribute to malignant phenotypes of glioma cells

Given that PMCAs belong to the P-type pump family, which is characterized by the formation of a high-energy acylphosphorylated intermediate during the reaction cycle [34], thus we hypothesize that ACYP2 may regulate intracellular Ca2+ homeostasis in glioma cells by hydrolyzing phosphoenzyme intermediate of PMCAs. In mammals, PMCAs are the products of four distinct genes (PMCA1–4), and the expression of different PMCA isoforms is regulated in a developmental, tissue- and cell type-specific manner [35]. Thus, we first determined mRNA levels of PMCA1–4 in a panel of glioma cell lines by qRT-PCR assay, and found relatively high expression of PMCA1 and 4 in most of cell lines (Additional file 2: Figure S11). In addition, there is evidence indicating that the remodeling of PMCA4 in colon cancer can promote proliferative pathways, while avoiding increased sensitivity to apoptotic stimuli [36]. Thus, we first knocked down PMCA4 using two different siRNAs (si-PMCA4–1023 and − 2728) in U251 and SF295 cells, and found that PMCA4 knockdown significantly inhibited cell proliferation in comparison with the control (Fig. 6a and b). Next, we performed Co-IP assay to confirm the interaction between ACYP2 and PMCA4 in the above cells (Fig. 6c). In addition, our data also demonstrated that the promoting effect of ACYP2 on cell proliferation and the activities of c-Myc and STAT3 could be clearly reversed by PMCA4 knockdown (Fig. 6d and e).

Based on the above findings, we propose a simple model to illustrate the mechanism underlying oncogenic role of ACYP2 in glioma (Fig. 6f). Briefly, ACYP2 interacts with and activates PMCA4 to promote Ca^2+^ efflux through hydrolyzing its phosphoenzyme intermediate. This will lead to decreased activity of calpain and subsequent activation of c-Myc and STAT3, thereby promoting malignant phenotypes of glioma cells.

### ACYP2 enhances tumorigenic potential in nude mice

**Fig. 7:**
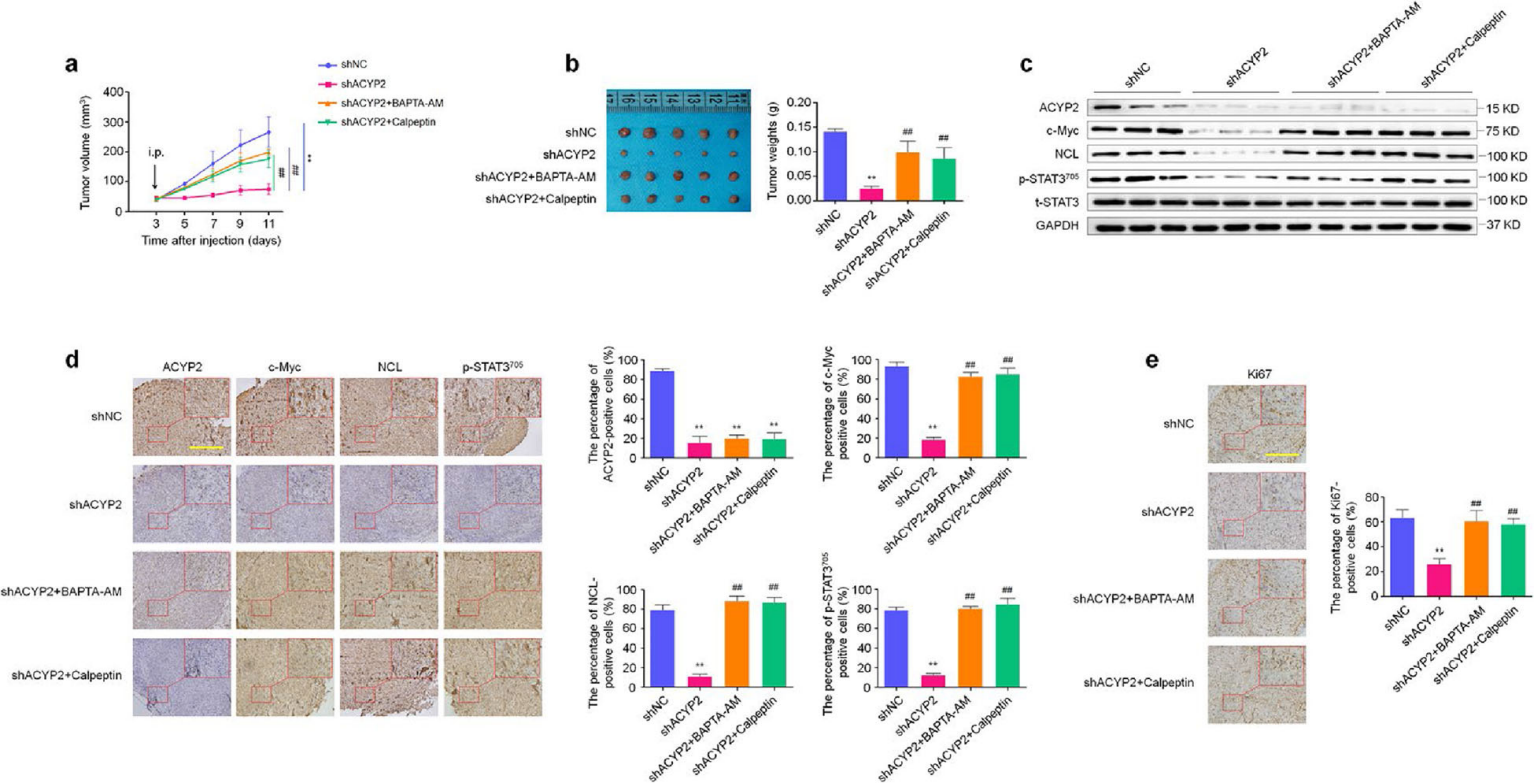
ACYP2 promotes tumor growth in nude mice. **a**, Growth curves of different xenograft tumors during the administration. Data are shown as mean ± SD (*n* = 5/group). Day 0th represents time point of tumor cell injection, and day 3th represents the beginning of dosing by intraperitoneal injection. **b**, Left and right panels show photographs of dissected tumors and mean tumor weight from the indicated groups, respectively. **c**, Western blot analysis was performed to analyze the levels of the indicated proteins in the xenigraft tumors from representative mice. GAPDH was used as a loading control. **d**, Representative sections from the indicated groups were subjected to IHC staining using corresponding antibodies. Histogram represents mean ± SD of the percentage of ACYP2, c-Myc, NCL and p-STAT3^705^-positive cells from five microscopic fields in each group, as shown in right panel. **e**, Shown is representative Ki67 staining of xenograft tumors from the indicated groups (left panel). Histogram represents mean ± SD of the percentage of Ki67 positive cells from five microscopic fields in each group, as shown in right panel. Scale bar, 200 μm. **, *P* < 0.01 for comparison with shNC; ^##^, *P* < 0.01 for comparison with shACYP2 + BAPTA-AM or Calpeptin

We also evaluated tumorigenic potential of ACYP2 in nude mice. Our data showed that the tumors induced by SF295 cells stably knocking down ACYP2 showed a much slower growth rate and smaller mean tumor volume than the tumors induced by control cells, while this effect could be clearly reversed by BAPTA-AM or calpeptin treatment (Fig. 7a and b). Next, we performed western blot analysis in xenograft tumors to further confirm the effect of ACYP2 on the activities of c-Myc and STAT3 in vivo. As shown in Fig. 7c, ACYP2 expression was significantly down-regulated in the ACYP2-knockdown tumors compared to control tumors; meanwhile, we also found that the levels of transcriptionally active c-Myc and its downstream target NCL, and STAT3 phosphorylation were dramatically decreased in the former relative to the latter, and this effect was similarly reversed by BAPTA-AM or calpeptin treatment. This was also supported by IHC assays (Fig. 7d). In addition, IHC results showed that ACYP2 knockdown leaded to low proliferative capacity of xenograft tumors relative to the control, as reflected by decreased percentage of Ki-67 positive cells, and this effect was similarly reversed by BAPTA-AM or calpeptin treatment (Fig. 7e). Collectively, these findings further support oncogenic role of ACYP2 in glioma cells by modulating intracellular Ca^2+^ homeostasis.

## Discussion

ACYP has been reported to be expressed exclusively in human metastatic colorectal cancer cells, suggesting that it may be involved in the metastatic phenotype [13]. However, its exact role in human cancers including glioma has not been elucidated. In this study, we provided strong evidence supporting that ACYP2 is a potent oncogene in glioma. First, we demonstrated that ACYP2 was significantly elevated in glioma tissues in comparison with control subjects, and found a significant association of increased expression of ACYP2 with poor survival in low-grade glioma patients. Second, ACYP2 knockdown clearly inhibited cell proliferation, colony formation, migration, invasion, and tumorigenic potential in nude mice, and induced cell apoptosis. On the other hand, ectopic expression of ACYP2 dramatically promoted malignant phenotypes of glioma cells, further supporting its oncogenic function.

To better understand biological role of ACYP2 in glioma, we first tested its effect on intracellular Ca^2+^ homeostasis in glioma cells. Our results showed that ACYP2 depletion led to a significant increase of intracellular Ca^2+^ concentration in the cytoplasm of tumor cells. To determine the role of intracellular Ca^2+^ homeostasis in tumor-promoting effect of ACYP2, we treated glioma cells with a highly selective Ca^2+^ chelator BAPTA-AM, and demonstrated that Ca^2+^ chelation could effectively reverse inhibitory effect of ACYP2 depletion on cell proliferation and migration. It is well known that calpains are a conserved family of cytosolic cysteine proteinases and their enzymatic activities depend on Ca^2+^. Members of the calpain family are implicated in several fundamental physiological processes, including cytoskeletal remodeling, apoptosis and cell survival [37–39]. In addition, aberrant expression of calpain has been implicated in tumorigenesis [40, 41]. Thus, we suppose that ACYP2 promotes malignant progression of glioma through modulating the activity of Ca2 + −dependent calpains. In this study, we expectedly found that ACYP2 knockdown caused a significant increase of calpain activity in glioma cells, and calpain inhibitor calpeptin could reverse inhibitory effect of ACYP2 knockdown on cell proliferation and migration, further proving the above hypothesis.

Considering the relatively poor specificity of calpain, we speculate that ACYP2 may act on some key transcription factors associated with malignant progression of glioma through regulating Ca2+/calpain signaling. There are studies showing that calpain inactivates the transcriptional function of c-Myc by removing its C-terminus [28, 42]. In this study, we expectedly found that ectopic expression of ACYP2 elevated the levels of transcriptionally active c-Myc and its downstream target NCL, while knocking down ACYP2 decreased their levels. This could be effectively reversed upon BAPTA-AM or calpeptin treatment.

In addition, STAT3 activation is also commonly found in human cancers including gliomas through modulating genes involved in cell growth, apoptosis, migration and invasion [43–45]. Notably, a previous study has revealed that EGF-induced STAT3 phosphorylation is highly Ca2 + −dependent, and partially regulated by the Ca2 + −permeable ion channel TRPM7 [31]. Given the above observations, we speculate that ACYP2 may affect phosphorylation and activity of STAT3 in glioma cells through regulating intracellular Ca2+ homeostasis. Indeed, our data showed that ACYP2 depletion dramatically decreased the levels of STAT3 phosphorylation in comparison with the control, and this could be reversed upon BAPTA-AM or calpeptin treatment. Given that there is no evidence showing direct interaction between calpain and STAT3, thus we speculate that there may exist certain intermediate molecules between them, which is involved in regulating STAT3 phosphorylation. Evidently, PTP1B can be cleaved by the Ca2 + −dependent neutral protease calpain in both platelet agonist-induced aggregation and intestinal epithelial cells [33, 46], and troglitazone, a PTP1B activator, can inhibit STAT3 phosphorylation and augment p53 expression in chronic lymphocytic leukemia (CLL) B cells [47]. It should be noted that PTP1B plays a critical role in down-regulation of activated STAT3 in glioma cells [48]. Thus, we suppose that ACYP2 may activate STAT3 through regulating calpain/PTP1B signal. This was proven by our data that Na3VO4, a non-specific PTP1B inhibitor, and siRNA targeting PTP1B could reverse inhibitory effect of ACYP2 depletion on STAT3 phosphorylation and cell proliferation.

For non-excitable cells, intracellular Ca2+ concentration is maintained at low levels mainly by ligand-gated channels (LGCs), store-operated channels (SOCs), receptor-operated calcium channels (ROCs), store-operated calcium entry (SOCE), stretch-activated channels (SACs), Na + −Ca2+ exchanger (NCX) and plasma membrane Ca2 + −ATPase (PMCA) [19]. The PMCAs belong to the P-type pump family, for which the binding of cytoplasmic Ca2+ is followed by formation of a “high energy” phosphoenzyme intermediate, commonly described as E1 and E2 states [49]. First, the E1-state enzyme exposes the high affinity Ca2+ binding site to the cytoplasmic side of the plasma membrane, followed by the conformational change of the enzyme to E2 state which induced by the phosphorylation of an invariant aspartate by ATP. Next, the enzyme exposes the bound Ca2+ to the extracellular side, lowers their affinity and release Ca2+. Finally, the phosphoenzyme intermediate is hydrolyzed and the enzyme returns to E1 state. In the present study, we find that ACYP2 functions as an oncogene in glioma cells through the interaction with PMCA4. The hydrolytic activity of ACYP2 on the phosphoenzyme intermediate of PMCA4 may accelerate the conformational change between E1 and E2, thereby increasing the efficiency of the transport and Ca2+ efflux.

## Conclusion

In summary, we find that increased expression of ACYP2 is frequently found in gliomas, and closely associated with poor patient survival. A series of in vitro and in vivo functional studies demonstrate that ACYP2 is a potent oncogene in glioma cells through promoting Ca^2+^ efflux and subsequently activating c-Myc and STAT3 signals. These data indicate that ACYP2 may be a potential therapeutic target and prognostic biomarker for glioma.

## Supplementary information

**Additional file 1: Table S1.** The primers used in this study for RT-PCR or qRT-PCR assays. **Table S2.** The short tandem repeat (STR) profiles of human glioma cell lines used in this study. **Table S3.** The sequences of siRNAs/shRNAs used in this study. **Table S4.** The primers used in this study for plasmid construction. **Table S5.** The antibodies used in this study.

**Additional file 2: Figure S1.** ACYP2 knockdown in glioma cells were confirmed by qRT-PCR assay. **Figure S2.** ACYP2 knockdown inhibits migration and invasion abilities of glioma cells. **Figure S3.** Ectopic expression of ACYP2 in SHG44 and A172 cells promoted cell migration and invasion compared to the control. **Figure S4.** The effect of ACYP2 knockdown on Ca^2+^ levels in endoplasmic reticulum (ER) in glioma cells. **Figure S5.** BAPTA-AM treatment reverses inhibitory effect of ACYP2 depletion on glioma cell migration. **Figure S6.** Calpeptin treatment reverses inhibitory effect of ACYP2 depletion on glioma cell migration. **Figure S7.** The effect of ACYP2 knockdown on the activity of NFATc1 in glioma cells. **Figure S8.** qRT-PCR was used to determine the effect of ACYP2 knockdown on the expression of NF-kB’s downstream targets (Bcl-xL and Bcl-2) in the indicated cells. **Figure S9.** qRT-PCR was used to determine the effect of ACYP2 knockdown on the expression of c-Myc’s downstream targets (E2F2 and cyclin E) and p-STAT3’s downstream targets (c-Fos and c-Jun). **Figure S10.** Cells transfected with the indicated constructs were treated with the vehicle or Stattic, and the MTT assay was then carried out to evaluate their effect on cell proliferation. **Figure S11.** qRT-PCR assay was performed to determine mRNA expression levels of PMCA1–4 in the indicated glioma cell lines.

## Data Availability

All data generated or analyzed during this study are included in this article.

## Abbreviations

ACYP2: Acylphosphatase 2
GBM: Glioblastoma
LGG: Low-grade glioma
STAT3: Signal transducer and activator of transcription 3
PTP1B: Protein tyrosine phosphatase 1B
CLL: Chronic lymphocytic leukemia
LGCs: Ligand-gated channels
SOCs: Store-operated channels
ROCs: Receptor-operated calcium channels
SOCE: Store-operated calcium entry
SACs: Stretch-activated channels
NCX: Na^+^-Ca^2+^ exchanger
PMCA: Plasma membrane Ca^2+^-ATPase
TCGA: The Cancer Genome Atlas
FBS: Fetal bovine serum
STR: Short tandem repeat
ORF: Open reading frame
MOI: Multiplicity of infection
RFU: Relative fluorescence units
SPF: Specific pathogen-free
qRT-PCR: quantitative RT-PCR
IHC: Immunohistochemistry
Co-IP: Co-immunoprecipitation
