# Supplementary material for: ACYP2 contributes to malignant progression of glioma through promoting Ca2+ efflux and subsequently activating c-Myc and STAT3 signals
Source: J Exp Clin Cancer Res. 2020 Jun 9;39:106. doi: 10.1186/s13046-020-01607-w (PMC7285537; doi:10.1186/s13046-020-01607-w)
Supplement: Supplementary file 1 — Additional file 1: Table S1. The primers used in this study for RT-PCR or qRT-PCR assays. Table S2. The short tandem repeat (STR) profiles of human glioma cell lines used in this study. Table S3. The sequences of siRNAs/shRNAs used in this study. Table S4. The primers used in this study for plasmid construction. Table S5. The antibodies used in this study. [file 13046_2020_1607_MOESM1_ESM.docx]

**Table S1.** The primers used in this study for RT-PCR or qRT-PCR assays

| **Genes** | **Forward primer (5’-3’)** | **Reverse primer (5’-3’)** |
| --- | --- | --- |
| *ACYP2* | TTGGCTGGGTGAAGAATACC | CTAGGGCTTCCAACCTTGCT |
| *NFATc1* | CCAGTCCCTTCCAAGTTTCCA | GACGTTGGAGGATGCATAGC |
| *NFATc2* | ACAGATCCCCCTTTCCAAACA | TGGCGACCTTATGTGCATTC |
| *NFATc3* | AGTGTGCTCTCATGTCCAGC | ATCCAGGGACTCAGGACTCG |
| *NFATc4* | TTCCCGGAAACTCCAGTCCA | CCTTTTCCTCCCCGAACACC |
| *PTGS2* | GTTCCACCCGCAGTACAGAA | AGGGCTTCAGCATAAAGCGT |
| *Bcl-xL* | TTCAGTGACCTGACATCCCA | CAGGAACCAGCGGTTGAA |
| *Bcl-2* | ATGTGTGTGGAGAGCGTCAA | GGGCCGTACAGTTCCACAAA |
| *NCL* | ACTGACCGGGAAACTGGGTC | TGGCCCAGTCCAAGGTAACT |
| *E2F2* | AGCTCATTGCCAAGAAGTCCA | GTCAACCCCTCAAGCCGTC |
| *cyclin E* | ATGTGTGTGGAGAGCGTCAA | GGGCCGTACAGTTCCACAAA |
| *c-Fos* | CCGGGGATAGCCTCTCTTAC | GTGACCGTGGGAATGAAGTT |
| *c-Jun* | TAACAGTGGGTGCCAACTCA | TTTTCTCTCCGTCGCAACTT |
| *PMCA1* | TACCTGAGGAGGAATTAGCAGAGGA | GTGGGCTTCCTGGGGATGAAGAGGT |
| *PMCA2* | AAGATCCACGGCGAGCGCAAT | GCTCGAGTTCTGCTTGAGCGC |
| *PMCA3* | GAAGACCTCACCCACAGAGG | TCTGCTCCTGCTCAATTCGG |
| *PMCA4* | TTACTCTCTTGGGGGTCAATGA | CCATGGTCTGCGATTTATCACA |
| *18S* | CGGCGCTAGAAGTGACATTC | CTTACGCGCTGGTCAGTGTT |
| *β-actin* | GCACAGAGCCTCGCCTT | CCTTGCACATGCCGGAG |

**Table S2.** The short tandem repeat (STR) profiles of human glioma cell lines used in this study

| **Cell lines** | **Markers** | |
| --- | --- | --- |
| U87 | [Amelogenin](https://strbase.nist.gov/Amelogenin.htm) | X (AddexBio; CCRID; CLS; ECACC); X,Y |
|  | [CSF1PO](https://strbase.nist.gov/str_CSF1PO.htm) | 10,11 |
|  | [D2S1338](https://strbase.nist.gov/str_D2S1338.htm) | 20,23 |
|  | [D3S1358](https://strbase.nist.gov/str_D3S1358.htm) | 16,17 |
|  | [D5S818](https://strbase.nist.gov/str_D5S818.htm) | 11,12 |
|  | [D7S820](https://strbase.nist.gov/str_D7S820.htm) | 8,9 |
|  | [D8S1179](https://strbase.nist.gov/str_D8S1179.htm) | 10,11 |
|  | [D13S317](https://strbase.nist.gov/str_D13S317.htm) | 8,11 |
|  | [D16S539](https://strbase.nist.gov/str_D16S539.htm) | 12 |
|  | [D18S51](https://strbase.nist.gov/str_D18S51.htm) | [13;13,14](https://www.ncbi.nlm.nih.gov/pubmed/22570425) |
|  | [D19S433](https://strbase.nist.gov/str_D19S433.htm) | 15,15.2 |
|  | [D21S11](https://strbase.nist.gov/str_D21S11.htm) | 28,32.2 |
|  | [FGA](https://strbase.nist.gov/str_FGA.htm) | 18,24 |
|  | [Penta D](https://strbase.nist.gov/str_Penta_D.htm) | 9,14 |
|  | [Penta E](https://strbase.nist.gov/str_Penta_E.htm) | 7,14 |
|  | [TH01](https://strbase.nist.gov/str_TH01.htm) | 9.3 |
|  | [TPOX](https://strbase.nist.gov/str_TPOX.htm) | 8 |
|  | [vWA](https://strbase.nist.gov/str_VWA.htm) | 15,17 |
| U251 | [Amelogenin](https://strbase.nist.gov/Amelogenin.htm) | X,Y; X |
|  | [CSF1PO](https://strbase.nist.gov/str_CSF1PO.htm) | 11,12; 12,13 |
|  | [D2S1338](https://strbase.nist.gov/str_D2S1338.htm) | 22,24 |
|  | [D3S1358](https://strbase.nist.gov/str_D3S1358.htm) | 16,17 |
|  | [D5S818](https://strbase.nist.gov/str_D5S818.htm) | 11,12; 11 |
|  | [D7S820](https://strbase.nist.gov/str_D7S820.htm) | 10,12 |
|  | [D8S1179](https://strbase.nist.gov/str_D8S1179.htm) | 13,15 |
|  | [D13S317](https://strbase.nist.gov/str_D13S317.htm) | 10,11 |
|  | [D16S539](https://strbase.nist.gov/str_D16S539.htm) | 12 |
|  | [D18S51](https://strbase.nist.gov/str_D18S51.htm) | 13 |
|  | [D19S433](https://strbase.nist.gov/str_D19S433.htm) | 13,15 |
|  | [D21S11](https://strbase.nist.gov/str_D21S11.htm) | [29,30; 29](https://www.ncbi.nlm.nih.gov/pubmed/27582061) |
|  | [FGA](https://strbase.nist.gov/str_FGA.htm) | 21,25; 20,21,25 |
|  | [Penta D](https://strbase.nist.gov/str_Penta_D.htm) | [10,12; 12](https://www.ncbi.nlm.nih.gov/pubmed/27582061) |
|  | [Penta E](https://strbase.nist.gov/str_Penta_E.htm) | 7,10; 7 |
|  | [TH01](https://strbase.nist.gov/str_TH01.htm) | 9.3 |
|  | [TPOX](https://strbase.nist.gov/str_TPOX.htm) | 8 |
|  | [vWA](https://strbase.nist.gov/str_VWA.htm) | 16,18 |
| SF295 | [Amelogenin](https://strbase.nist.gov/Amelogenin.htm) | X |
|  | [CSF1PO](https://strbase.nist.gov/str_CSF1PO.htm) | 10,13 |
|  | [D2S1338](https://strbase.nist.gov/str_D2S1338.htm) | 15,20 |
|  | [D3S1358](https://strbase.nist.gov/str_D3S1358.htm) | 16,18 |
|  | [D5S818](https://strbase.nist.gov/str_D5S818.htm) | 11,12 |
|  | [D7S820](https://strbase.nist.gov/str_D7S820.htm) | 9,13 |
|  | [D8S1179](https://strbase.nist.gov/str_D8S1179.htm) | 13,14 |
|  | [D13S317](https://strbase.nist.gov/str_D13S317.htm) | 10 |
|  | [D16S539](https://strbase.nist.gov/str_D16S539.htm) | 12,13 |
|  | [D18S51](https://strbase.nist.gov/str_D18S51.htm) | 15,18 |
|  | [D19S433](https://strbase.nist.gov/str_D19S433.htm) | 12,15 |
|  | [D21S11](https://strbase.nist.gov/str_D21S11.htm) | 28,30 |
|  | [FGA](https://strbase.nist.gov/str_FGA.htm) | 22,25 |
|  | [TH01](https://strbase.nist.gov/str_TH01.htm) | 9.3; 9.3,10 |
|  | [TPOX](https://strbase.nist.gov/str_TPOX.htm) | 8,11 |
|  | [vWA](https://strbase.nist.gov/str_VWA.htm) | 16,17 |
| A172 | [Amelogenin](https://strbase.nist.gov/Amelogenin.htm) | X,Y |
|  | [CSF1PO](https://strbase.nist.gov/str_CSF1PO.htm) | 9,12; 9,11 |
|  | [D1S1656](https://strbase.nist.gov/str_D1S1656.htm) | 12,14 |
|  | [D2S1338](https://strbase.nist.gov/str_D2S1338.htm) | 20,21 |
|  | [D3S1358](https://strbase.nist.gov/str_D3S1358.htm) | 14,18; 15,18 |
|  | [D5S818](https://strbase.nist.gov/str_D5S818.htm) | 11,12 |
|  | [D6S1043](https://strbase.nist.gov/str_D6S1043.htm) | 13,18 |
|  | [D7S820](https://strbase.nist.gov/str_D7S820.htm) | 11 |
|  | [D8S1179](https://strbase.nist.gov/str_D8S1179.htm) | 13,14 |
|  | [D12S391](https://strbase.nist.gov/strbase/str_D12S391.htm) | 22 |
|  | [D13S317](https://strbase.nist.gov/str_D13S317.htm) | 11 |
|  | [D16S539](https://strbase.nist.gov/str_D16S539.htm) | 12;11 |
|  | [D18S51](https://strbase.nist.gov/str_D18S51.htm) | 12,13 |
|  | [D19S433](https://strbase.nist.gov/str_D19S433.htm) | 12,15.2 |
|  | [D21S11](https://strbase.nist.gov/str_D21S11.htm) | 28,32.2; 27,32 |
|  | [FGA](https://strbase.nist.gov/str_FGA.htm) | 20,22 |
|  | [Penta D](https://strbase.nist.gov/str_Penta_D.htm) | 9,13 |
|  | [Penta E](https://strbase.nist.gov/str_Penta_E.htm) | 5,10 |
|  | [TH01](https://strbase.nist.gov/str_TH01.htm) | 6,9.3; 6,10 |
|  | [TPOX](https://strbase.nist.gov/str_TPOX.htm) | 8,11 |
|  | [vWA](https://strbase.nist.gov/str_VWA.htm) | 20; 16,20; 17,20 |

**Table S3.** The sequences of siRNAs/shRNAs used in this study

| **siRNAs/shRNAs** | **Sense (5’-3’)** | **Antisense (5’-3’)** |
| --- | --- | --- |
| si-ACYP2-482 | GGUGUUUGCUUCAGAAUGUTT | ACAUUCUGAAGCAAACACCTT |
| si-ACYP2-540 | GGGUGAAGAAUACCAGCAATT | UUGCUGGUAUUCUUCACCCTT |
| si-PMCA4-1023 | AAGACCAAGGAUGGCGUGGUGTT | CACCACGCCAUCCUUGGTCUUTT |
| si-PMCA4-2728 | CUGCUUGUCUCCUACUAUATT | UAUAGUAGGAGACAAGCAGTT |
| si-PTP1B | CUUCCGUUGAUAUCAAGAATT | UUCUUGAUAUCAACGGAAGTT |
| si-NC | UUCUCCGAACGUGUCACGUTT | ACGUGACACGUUCGGAGAATT |
| shACYP2 | GATCCGGTGTTTGCTTCAGAATGTTTCAAGAGAACATTCTGAAGCAAACACCTTTTTTC | AATTGAAAAAAGGTGTTTGCTTCAGAATGTTCTCTTGAAACATTCTGAAGCAAACACCG |
| shNC | GATCCGTTCTCCGAACGTGTCACGTAATTCAAGAGATTACGTGACACGTTCGGAGAATTTTTTC | AATTCAAAAAATTCTCCGAACGTGTCACGTAATCTCTTGAATTACGTGACACGTTCGGAGAACG |

**Table S4.** The primers used in this study for plasmid construction

| **Constructs** | **Position** | **Forward primer (5’-3’)** | **Reverse primer (5’-3’)** | **Restriction sites** |
| --- | --- | --- | --- | --- |
| ACYP2 | CDS | AATTTCTCGAGGCCACCAACACGAGGCCGAATTCCAA | ATATAGAATTCCACGTACAGTCGCTTAGCTT | XhoI and EcoRI |

**Table S5.** The antibodies used in this study

| **Antibodies** | **Catalog#** | **Source** |
| --- | --- | --- |
| anti-IgG | ab6715 | Abcam |
| anti-ACYP2 | NBP1-86308 | Novus |
| anti-GAPDH | M20006 | Abmart |
| anti-Tubulin | sc-101527 | Santa Cruz |
| anti-β-actin | sc-8432 | Santa Cruz |
| anti-total STAT3 | AP0365 | Bioworld Technology |
| anti-phospho-STAT3^Y705^ | BS4181 | Bioworld Technology |
| anti-NFATc1 | sc-7294 | Santa Cruz |
| anti-Histone H3 | ab1791 | Abcam |
| anti-c-Myc | sc-764 | Santa Cruz |
| anti-NCL | sc-8031 | Santa Cruz |
| anti-PMCA4 | sc-20027 | Santa Cruz |
| anti-Ki67 | Cat550609 | BD Pharmingen |
