# Supplementary material for: ACYP2 contributes to malignant progression of glioma through promoting Ca2+ efflux and subsequently activating c-Myc and STAT3 signals
Source: J Exp Clin Cancer Res. 2020 Jun 9;39:106. doi: 10.1186/s13046-020-01607-w (PMC7285537; doi:10.1186/s13046-020-01607-w)
Supplement: Supplementary file 2 — Additional file 2: Figure S1. ACYP2 knockdown in glioma cells were confirmed by qRT-PCR assay. Figure S2. ACYP2 knockdown inhibits migration and invasion abilities of glioma cells. Figure S3. Ectopic expression of ACYP2 in SHG44 and A172 cells promoted cell migration and invasion compared to the control. Figure S4. The effect of ACYP2 knockdown on Ca2+ levels in endoplasmic reticulum (ER) in glioma cells. Figure S5. BAPTA-AM treatment reverses inhibitory effect of ACYP2 depletion on glioma cell migration. Figure S6. Calpeptin treatment reverses inhibitory effect of ACYP2 depletion on glioma cell migration. Figure S7. The effect of ACYP2 knockdown on the activity of NFATc1 in glioma cells. Figure S8. qRT-PCR was used to determine the effect of ACYP2 knockdown on the expression of NF-kB’s downstream targets (Bcl-xL and Bcl-2) in the indicated cells. Figure S9. qRT-PCR was used to determine the effect of ACYP2 knockdown on the expression of c-Myc’s downstream targets (E2F2 and cyclin E) and p-STAT3’s downstream targets (c-Fos and c-Jun). Figure S10. Cells transfected with the indicated constructs were treated with the vehicle or Stattic, and the MTT assay was then carried out to evaluate their effect on cell proliferation. Figure S11. qRT-PCR assay was performed to determine mRNA expression levels of PMCA1–4 in the indicated glioma cell lines. [file 13046_2020_1607_MOESM2_ESM.docx]

**Figure S1.** ACYP2 knockdown by two different siRNAs (si-ACYP2-482 and -540) in U251, SF295 and U87 cells were confirmed by qRT-PCR assay. *18S* rRNA was used as a reference. **, *P* <0.01.

**Figure S2.** ACYP2 knockdown inhibits migration (**a**) and invasion (**b**) abilities of glioma cells. Left panels represent representative images of migrated/invaded cells. Quantitative illustration of cell numbers is shown as means ± SD in right panels. **, *P* <0.01; ***, *P* <0.001.

**Figure S3.** Ectopic expression of ACYP2 in SHG44 and A172 cells clearly promoted cell migration (**a**) and invasion (**b**) in comparison with the control. Left panels represent representative images of migrated/invaded cells. Quantitative illustration of cell numbers is shown as means ± SD in right panels. **, *P* <0.01

**
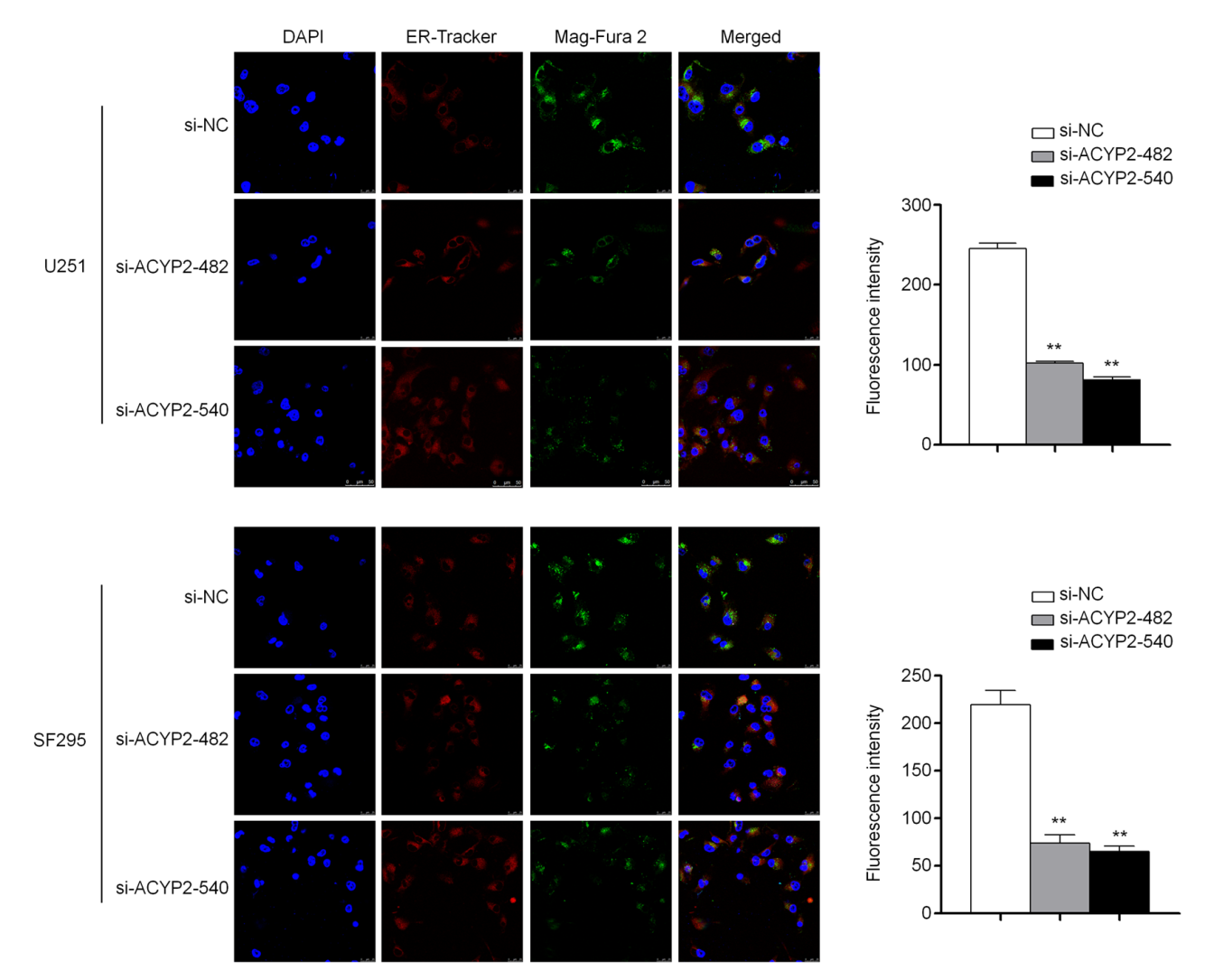
**

**Figure S4.** The effect of ACYP2 knockdown on Ca^2+^ levels in endoplasmic reticulum (ER) in glioma cells. Representative images showing Ca^2+^ levels in endoplasmic reticulum in the indicated cells under the confocal microscope (left panel). Blue color represents staining of nuclei. Red color represents the staining of endoplasmic reticulum. Green color represents staining of Ca^2+^ in the ER. Histogram represents mean ± SD of the fluorescence intensity from five microscopic fields in each group, as shown in right panel. Scale bars, 250 μm. **, *P* <0.01.

**
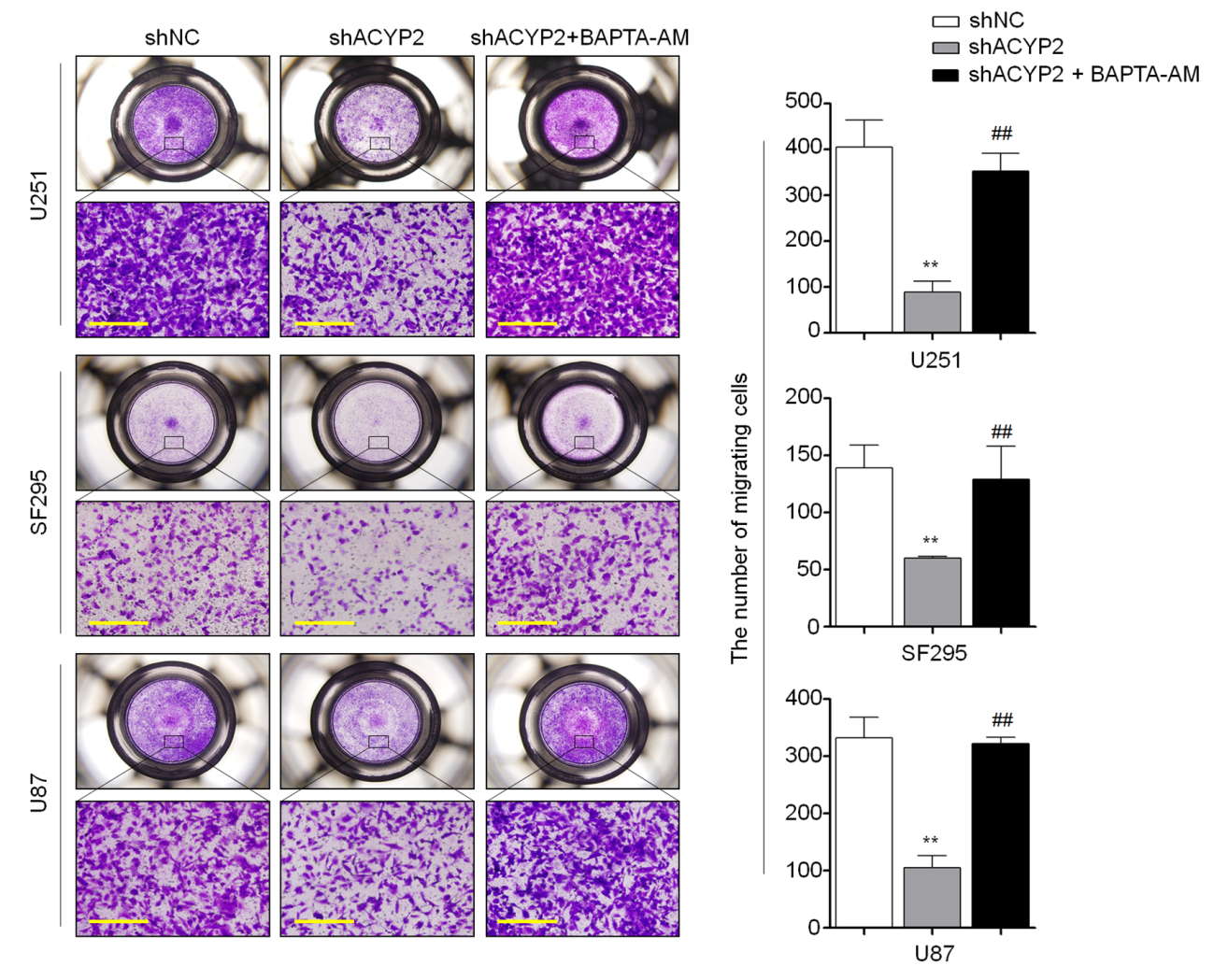
**

**Figure S5.** BAPTA-AM treatment reverses inhibitory effect of ACYP2 depletion on glioma cell migration. Cells stably knocking down ACYP2 or control cells were treated with the vehicle or 5 μM BAPTA-AM for 6 h. Representative images of migrated cells were shown in left panels. Histograms which corresponding to left panel showing means ± SD of cell numbers from five randomly selected high power field (right panel). **, *P* < 0.01 for comparison with shNC; ^##^, *P* < 0.01 for comparison with shACYP2+BAPTA-AM.

**
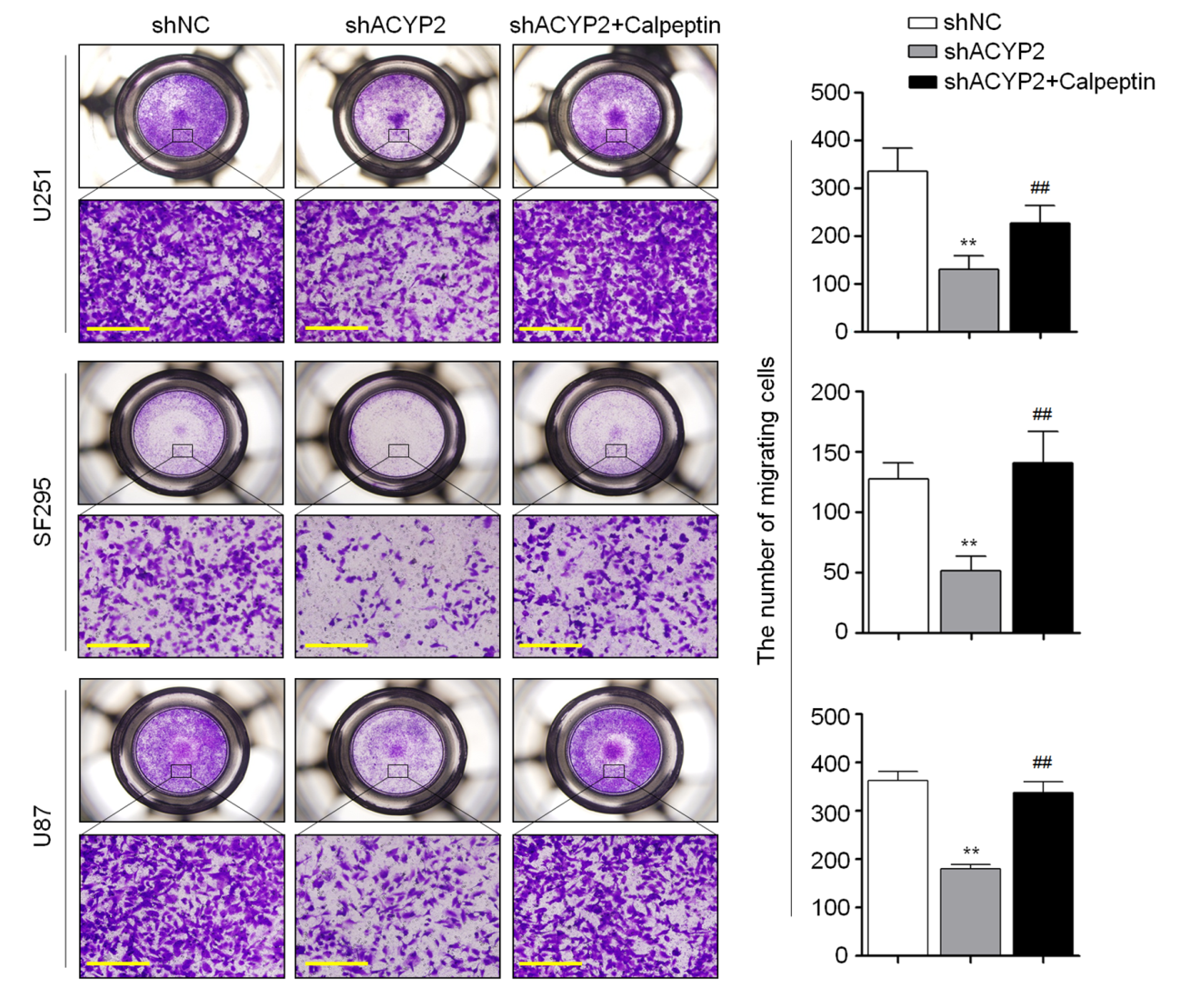
**

**Figure S6.** Calpeptin treatment reverses inhibitory effect of ACYP2 depletion on glioma cell migration. Cells stably knocking down ACYP2 or control cells were treated with the vehicle or 10 μM calpeptin for 12 h. Representative images of migrated cells were shown in left panels. Histograms which corresponding to left panel showing means ± SD of cell numbers from five randomly selected high power field (right panel). **, *P* < 0.01 for comparison with shNC; ^##^, *P* < 0.01 for comparison with shACYP2+Calpeptin.

**
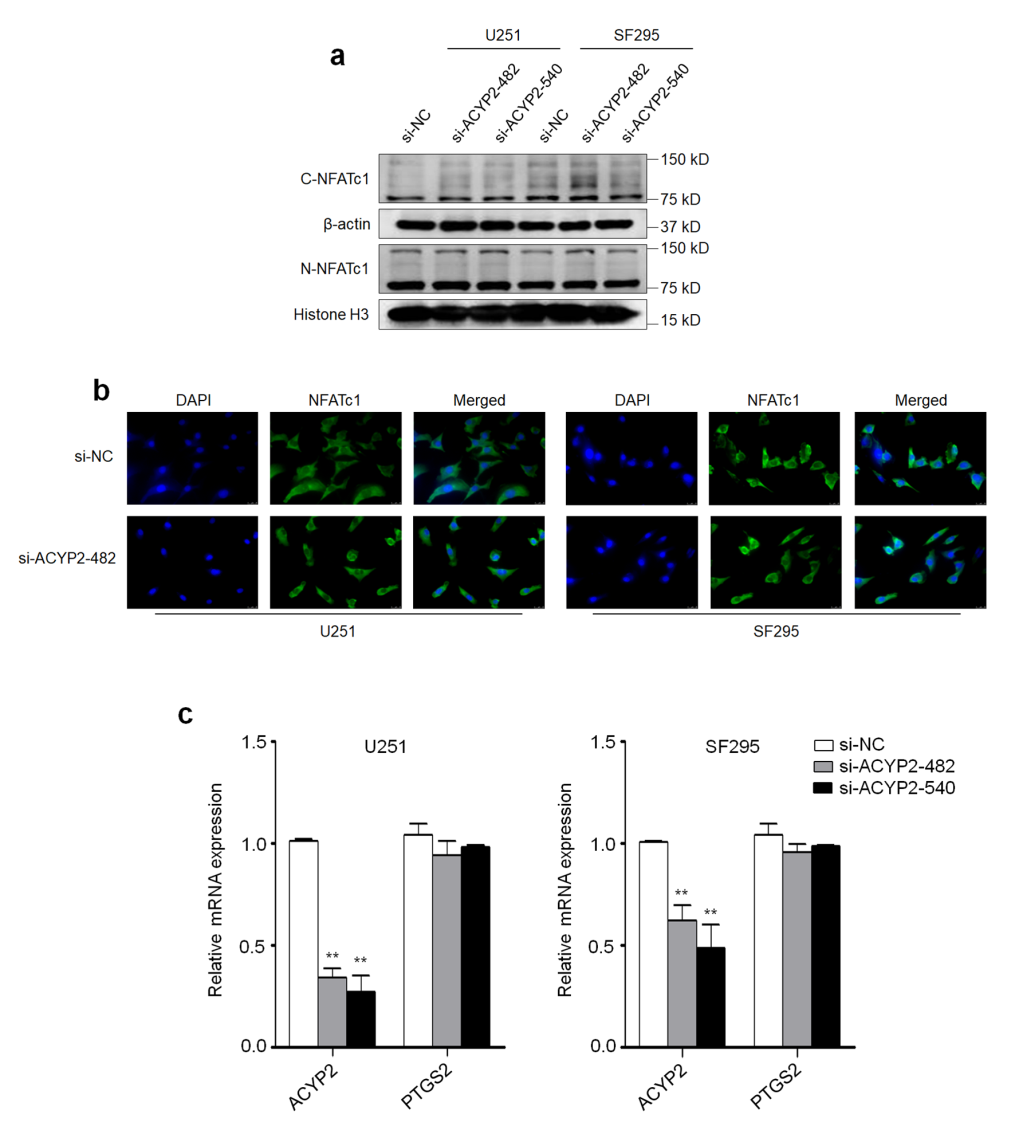
Figure S7.** The effect of ACYP2 knockdown on the activity of NFATc1. **a**, Cytosolic and nuclear fractions of the indicated cells were prepared and analyzed by western blot to determine the effect of ACYP2 knockdown on NFATc1 expression. β-actin and Histone H3 were used as loading controls. C-NFATc1, cytosolic NFATc1; N-NFATc1, nuclear NFATc1. **b**, The effect of ACYP2 knockdown on cytoplasm-to-nucleus translocation of NFATc1 in the indicated cells using a fluorescent microscope. **c**, qRT-PCR assay was used to evaluate the effect of ACYP2 knockdown on mRNA levels of NFATc1’s downstream target PTGS2 in the indicated cells. *18S* rRNA was used as a reference gene. **, *P* <0.01.

**
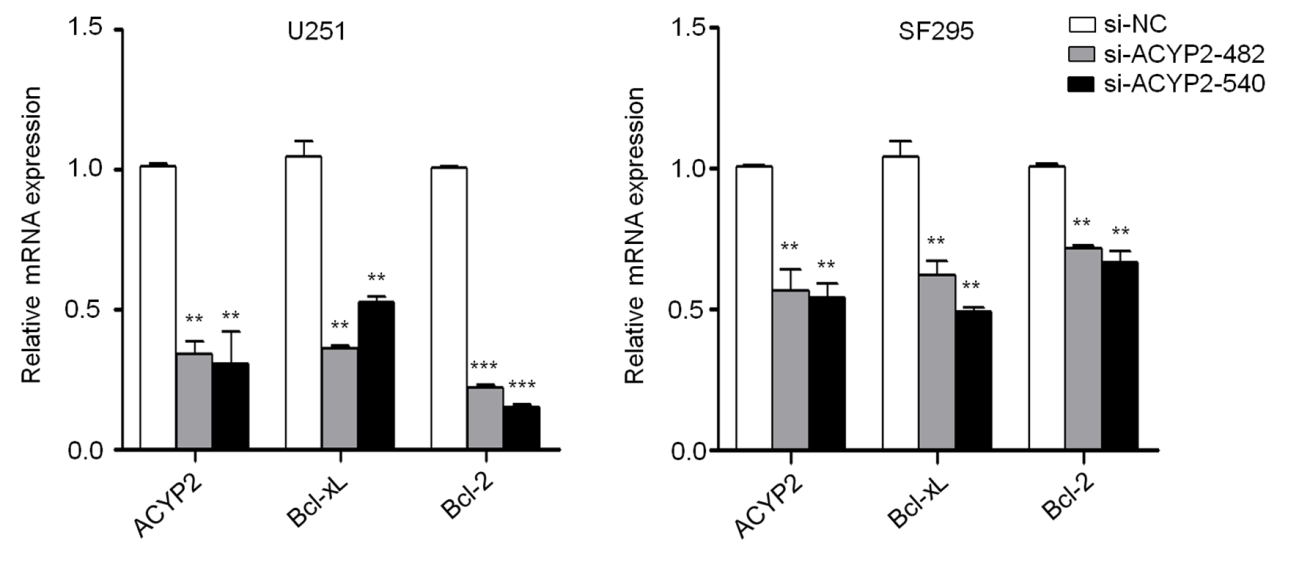
**

**Figure S8.** qRT-PCR was used to determine the effect of ACYP2 knockdown on the expression of NF-kB’s downstream targets (Bcl-xL and Bcl-2) in the indicated cells. *18S* rRNA was used as a reference gene. **, *P* <0.01; ***, *P* <0.001.


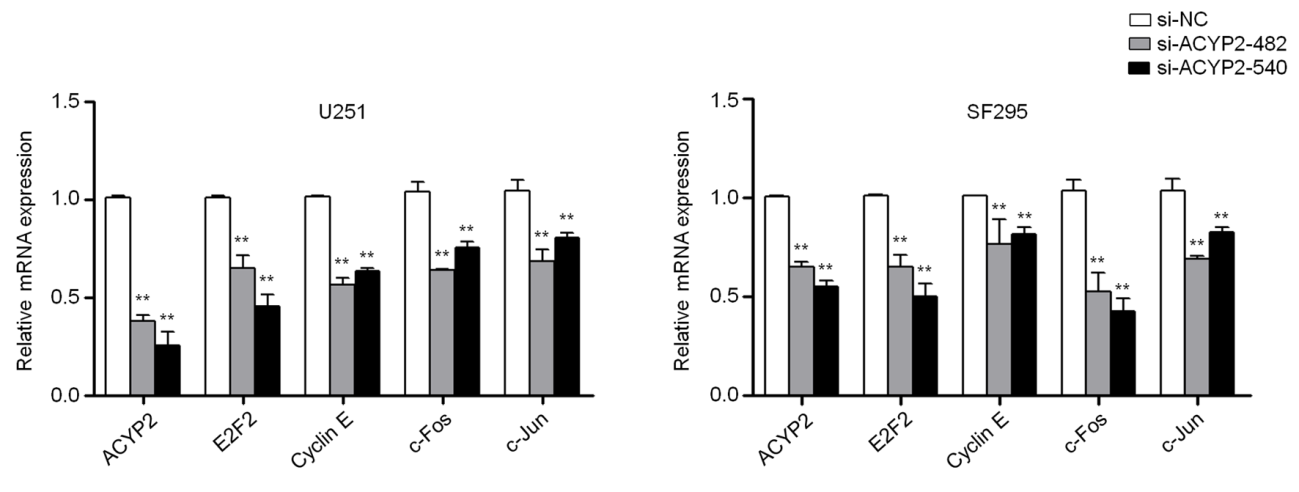


**Figure S9.** qRT-PCR was used to determine the effect of ACYP2 knockdown on the expression of c-Myc’s downstream targets (E2F2 and cyclin E) and p-STAT3’s downstream targets (c-Fos and c-Jun). *18S* rRNA was used as a reference gene. **, *P* <0.01.

**Figure S10.** Cells transfected with the indicated constructs were treated with the vehicle or 1 μM (or 5 μM) Stattic for 1 h, and the MTT assay was then carried out to evaluate their effect on cell proliferation. The data were presented as mean ± SD (n =3). **, *P* <0.01; ***, *P* <0.001.

**
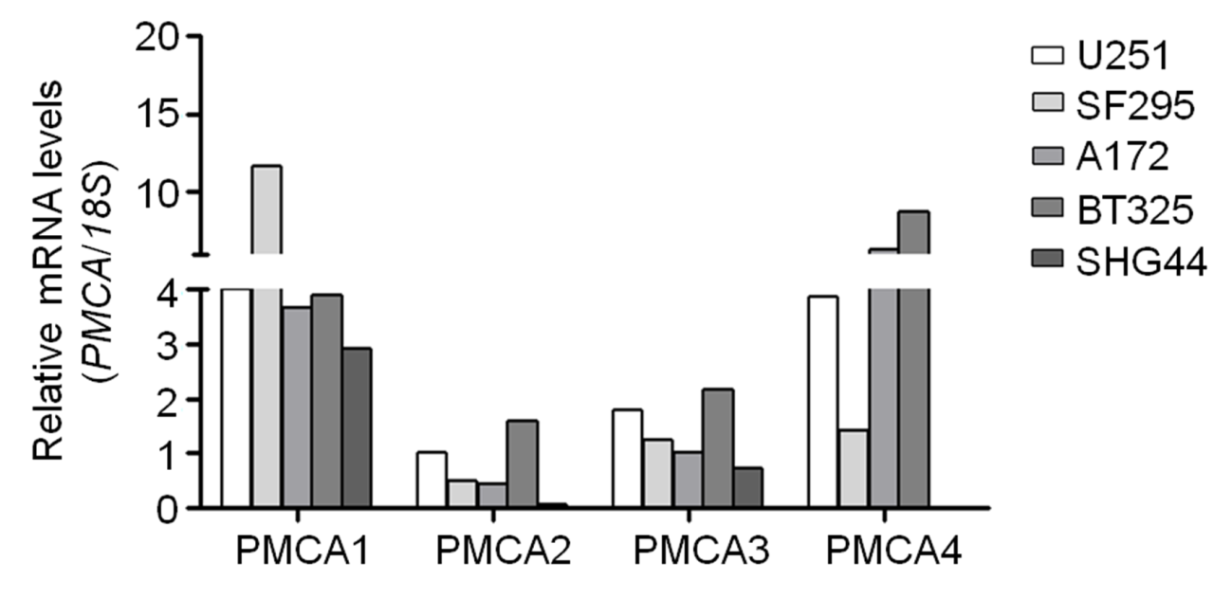
**

**Figure S11.** qRT-PCR assay was performed to determine mRNA expression levels of PMCA1-4 in the indicated cell lines. Their expression was normalized to *18S* rRNA levels.
